# Caring for high-need patients

**DOI:** 10.1186/s12913-023-10236-w

**Published:** 2023-11-23

**Authors:** Susanne Hempel, Maria Bolshakova, Michael Hochman, Elvira Jimenez, Gina Thompson, Aneesa Motala, David A. Ganz, Sonya Gabrielian, Samuel Edwards, James Zenner, Ben Dennis, Evelyn Chang

**Affiliations:** 1 Southern California Evidence Review Center, University of Southern California, Los Angeles, USA; 2 Gehr Family Center for Health Systems Science and Innovation, University of Southern California, Los Angeles, CA USA; 3 VA Greater Los Angeles Healthcare System, Los Angeles, CA USA; 4 VA Portland Health Care System, Portland, OR USA; 5 Los Angeles County Department of Mental Health, Los Angeles, CA USA

**Keywords:** High need patients, Environmental scan, Key informants, Expert panel, Care stakeholders

## Abstract

**Objective:**

We aimed to explore the construct of “high need” and identify common need domains among high-need patients, their care professionals, and healthcare organizations; and to describe the interventions that health care systems use to address these needs, including exploring the potential unintended consequences of interventions.

**Methods:**

We conducted a modified Delphi panel informed by an environmental scan. Expert stakeholders included patients, interdisciplinary healthcare practitioners (physicians, social workers, peer navigators), implementation scientists, and policy makers. The environmental scan used a rapid literature review and semi-structured interviews with key informants who provide healthcare for high-need patients. We convened a day-long virtual panel meeting, preceded and followed by online surveys to establish consensus.

**Results:**

The environmental scan identified 46 systematic reviews on high-need patients, 19 empirical studies documenting needs, 14 intervention taxonomies, and 9 studies providing construct validity for the concept “high need.” Panelists explored the construct and terminology and established that individual patients’ needs are unique, but areas of commonality exist across all high-need patients. Panelists agreed on 11 domains describing patient (e.g., social circumstances), 5 care professional (e.g., communication), and 8 organizational (e.g., staffing arrangements) needs. Panelists developed a taxonomy of interventions with 15 categories (e.g., care navigation, care coordination, identification and monitoring) directed at patients, care professionals, or the organization. The project identified potentially unintended consequences of interventions for high-need patients, including high costs incurred for patients, increased time and effort for care professionals, and identification of needs without resources to respond appropriately.

**Conclusions:**

Care for high-need patients requires a thoughtful approach; differentiating need domains provides multiple entry points for interventions directed at patients, care professionals, and organizations. Implementation efforts should consider outlined intended and unintended downstream effects on patients, care professionals, and organizations.

**Supplementary Information:**

The online version contains supplementary material available at 10.1186/s12913-023-10236-w.

## Introduction

High-need patients present challenges to healthcare delivery organizations due to their heterogeneity [1], complexity from multimorbidity or social circumstances, [2, 3] frequent healthcare utilization, [4] and need for higher levels of assistance [5]. These challenges exist at the patient (e.g., poor functional health), healthcare care professionals (e.g., limited training), and the healthcare organization (e.g., resources) level [6].

Many interventions addressing high-need patients have aimed to improve health and health care, to enhance experiences with healthcare, to decrease utilization, and ultimately, to decrease healthcare cost; but there are few clear solutions [7]. Health care systems that seek to improve healthcare delivery for high-need patients may benefit from a greater understanding of the concept of high-need as well as approaches that have been described in literature, including effects on the patients, their care professionals, and the health care organizations.

In this study, we combined an environmental scan with a stakeholder panel to understand the breadth of interventions that have been implemented for high-need patients in healthcare organizations. The environmental scan included insights from key informants and published literature. We engaged stakeholders in a modified Delphi panel process with the following objectives: (1) To explore the construct of “high need” and to identify common needs among high-need patients, their healthcare professionals, and healthcare organizations; and (2) to describe the interventions that health care professionals and health care systems use to address these needs, including exploring potential unintended consequences of the healthcare interventions. We aimed to develop a framework of needs and interventions that can be employed by health systems aiming to improve care for their most vulnerable patients.

## Materials and methods

A detailed workplan guided a one-year research process. An environmental scan consisted of a synthesis of published literature and key informant interviews. The results of this environmental scan informed an expert stakeholder panel. The University of Southern California Institutional Review board determined the study to be exempt.

### Environmental scan

Environmental scan sources included interviews with key informants and a review of published literature.

### Key informant interviews

We selected key informants to provide information potentially not yet available in the published research literature: An experienced social worker from the VA healthcare system; the evidence review team working on the Agency for Healthcare Research and Quality evidence report on high utilizers; [7] an emergency medicine physician in downtown Los Angeles who serves complex patients in underserved communities; and team members on an intensive case management demonstration program from the VA healthcare system team. Key informants provided written and verbal input. Verbal input was obtained in one-hour semi-structured phone interviews. We grouped responses by the definition of “high need,” the content of patient and health care professional needs, intervention types and unintended consequences, issues not fully recognized in research and policy, and available tools (Appendix Table 1).

In addition, we reviewed transcripts of qualitative interviews that were collected through a multi-site intensive case management pilot in primary care to improve care for high-need patients [8]. Appendix Table 2 documents quotes from health care professionals and are thematically organized by categories of needs: social determinants of health; substance use disorder, with or without co-occurring mental health issues; mental health; physical health; multimorbidity or medical complexity; chronic condition or disease management; patient or healthcare professional education; reliable access to healthcare professionals and services; patient or caregiver-clinician and team member relationships; trust; lifestyle change; changed outlook; difficulties in the home; improvements needed in the home environment; challenges navigating VHA care/services; and communication and care coordination. This in-depth analysis informed the stakeholder groups as well as topics that need to be explored further in the literature and panel discussions.

### Literature review

The literature review identified systematic reviews that addressed complex patients and empirical investigations aiming to determine the needs of complex primary care patients. We followed rapid literature review methodology and searched the research databases PubMed, CINHAL, and the Web of Science. Searches were designed and executed by an experienced librarian specializing in evidence reviews. Because standard nomenclature for high-need research does not exist, the complex concepts had to be translated into a comprehensive search strategy (see online appendix). In addition, we mined the references of relevant reviews. Two literature reviewers screened the search output, and all publications deemed potentially relevant by at least one reviewer were obtained as full text. The full-text publications were screened against transparent pre-specified eligibility criteria, and the inclusion and exclusion decisions were recorded together with the reasons for exclusion. Eligibility criteria included systematic reviews, empirical studies describing needs, studies validating the construct, intervention taxonomies, and tool collections; full details are provided in the appendix.

Literature searches identified 2,582 citations. Of these, 195 publications were obtained as full text, and 78 were included. The flow diagram (Appendix Fig. 1) provides an overview of the identified literature and reasons for exclusion for excluded studies.

**Fig. 1:**
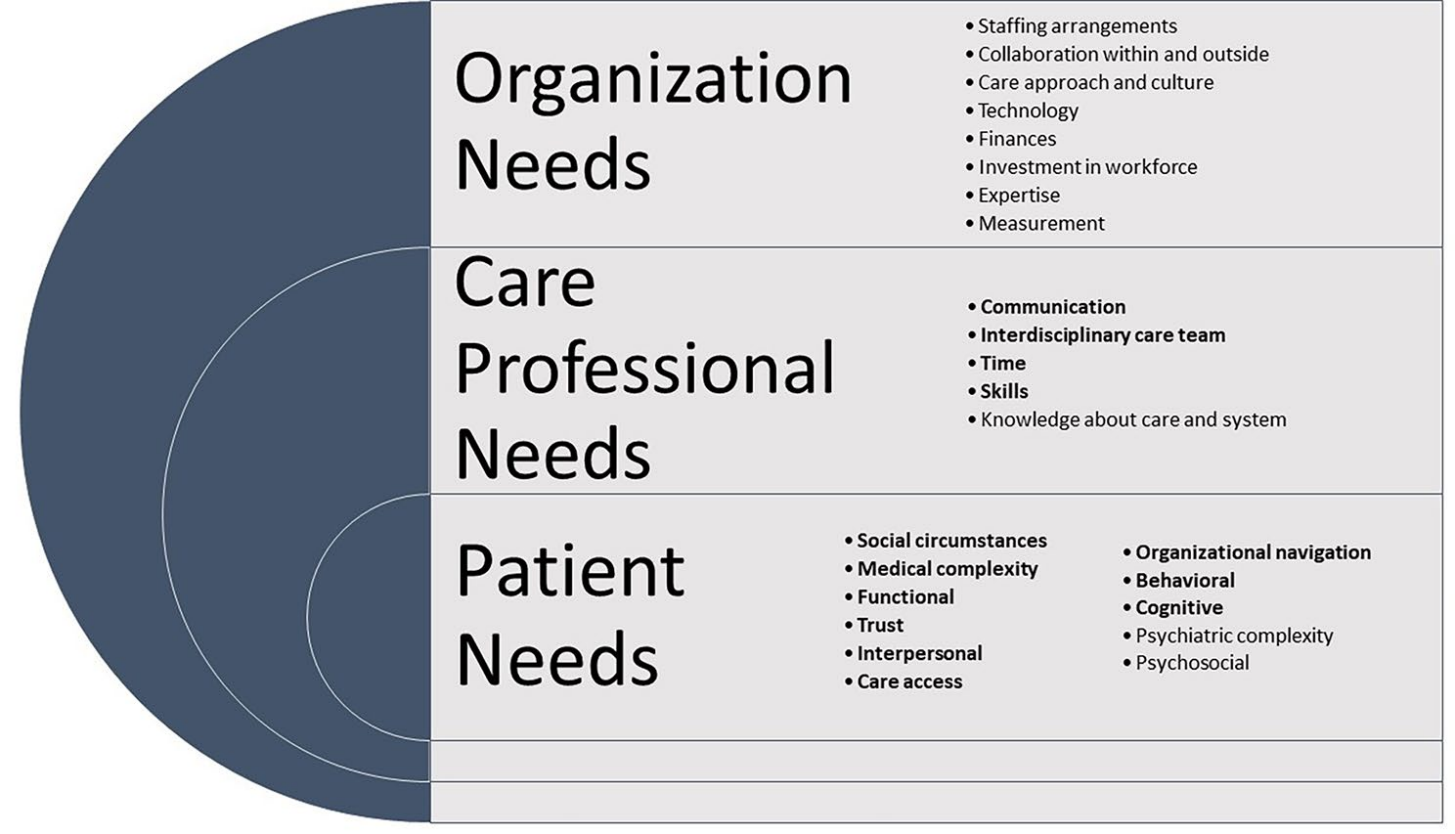
Need domains of high-needs patients, their healthcare professionals, and care organizations Note: Bolded domains were rated as essential, domains rated as important are also shown (not bolded)

### Stakeholder panel

We recruited a stakeholder panel representing diverse viewpoints. The panel recruiting process followed a structured, two-step approach: identifying key stakeholders by using a patient-centered framework, [9] followed by finding individuals who could represent the stakeholder group. The 12 panelists (see appendix) included patients, physicians and nurses experienced in the care of high-need patients, policy makers, social worker, peer navigator, and content experts in improvement and implementation science, all of whom were associated with large integrated healthcare delivery systems. The panelists also had clinical experience in four key areas for this patient population: mental health, homelessness, older adults, and addiction.

### Data collection

The consensus-finding process consisted of a pre- and post-panel online survey and a day-long virtual panel meeting with presentations and moderated discussions. Panelists were provided with the results of the environmental scan prior to completing the pre-panel survey. Our modified Delphi panel process adhered to principles of consensus methods for medical and health services research: anonymity (private ranking or voting to avoid dominance issues in the group), iteration (multiple rounds to allow individuals to change their opinions after discussions), controlled feedback (feedback of the group response after each rating round), and statistical group response (provision of summary measures of the group response) [10].

The pre-panel survey was divided into sections addressing procedures, terminology, and measurement or operationalization. Panelists were asked about guidance for high-need patients, how the organization defined high need, and how high-need patients were identified in their local organization. Panelists rated characteristics of high-need patients in order to establish a shared understanding of the concept. The survey assessed whether crosscutting recommendations regarding high-need patients are possible, given the heterogeneity of this patient group. Panelists rated 22 different statements regarding the generalizability of tools and recommendation (e.g., “as interventions may need to be uniquely targeted to individual patients, generic recommendations may not be possible.”) The survey asked panelists for a description of the needs of high-need patients and to rate components of need based on suggestions identified in the literature. In addition, about it targeted needs of primary care professionals caring for high-need patients that go beyond the routine strains of the care environment (e.g., clinician burnout is not specific to high-need patients). In addition, the survey elicited needs of the healthcare organizations caring for high-need patients. The survey asked respondents about unintended consequences of interventions for high-need patients that might affect patients, healthcare professionals, or healthcare organizations. Finally, panelists were given an opportunity to identify issues not sufficiently covered in research or existing policy, examples of successful approaches for high-need patients, information on interventions that have been implemented to address high-need patients in panelists’ local organizations, and helpful tools for high-need patients.

The one-day expert panel meeting focused on approaches for high-need patients. The panel meeting had been planned as an in-person meeting, following a standard procedure, [11–15] but had to be converted to an online virtual meeting due to the COVID-19 pandemic. The research team presented the results of the pre-panel survey. Panelists discussed the ratings, focusing primarily on areas of disagreement. Following an approach used for RAND appropriateness panels, no attempt was made to force the panel to consensus [16]. Instead, the process was designed to distinguish between discrepant ratings due to true disagreement (i.e., actual differences in opinion) and misunderstandings (i.e., interpretation differences that can be resolved). In addition to the 12 panelists, several observers were present. Observers were advised to not interrupt the discussions during the panel meeting, and they did not complete the pre-or post-panel survey nor did they vote during the panel meetings. Observers made valuable contributions using the chat function during the panel meeting by providing examples or additional discussion points.

After the meeting, a post-panel survey was sent to panelists to consolidate findings. The survey included pre-panel items that had been discussed during the panel meeting and new items generated in panel discussions. Specifically, the post-panel survey was completed after the meeting to avoid group pressure and groupthink, ensuring independent ratings.

All items were rated on a scale from 1 to 5, where five represented the highest level of importance or relevance, or *essential*. We analyzed the central tendency, dispersion in ratings, the mode, and the proportion of panelists judging the item to be essential. To determine disagreement, we used a standard deviation value of greater than one and situations where a small number of panelists (up to 30%) identified the item as essential while others did not rate the item as particularly important. Any item that had a mean value equal or above 3.5 across all panelists was considered *important*, values equal or above 4.5 *essential*.

## Results

The following describes results regarding the concept of high-need patients, domains of needs, and intervention characteristics.

### High need concept and terminology

The content of the key informant interviews is documented in detail (Appendix Table 1). All key informants indicated that there is no universally accepted definition of high-need patients, that research and policy often focus on specific patient populations (e.g., potentially preventable high use of healthcare), and that *high need* can mean unique circumstances for individual patients.

The evidence table (Appendix Table 3) summarizes the 46 identified research syntheses presented to the panelists [17–62]. The publications spanned over a decade (the earliest identified review searched the literature in 2006), but a third of the summaries were published in 2018 and 2019. Reviews described the populations most often as patients with multi-morbidity and complex patients. Others addressed research in high-need, high-cost populations; patients with multiple health and social care needs; high-need and high-risk patients; chronic diseases and complex healthcare needs; older participants with several chronic conditions; and patients with mental and physical multimorbidity. Reviews also operationalized the specific population of high-need patients differently, such as multimorbidity (e.g., defined as two or medical diagnoses or multiple chronic conditions), and noted that definitions and measurement vary across studies included in a review [43].

Seven reviews provided construct validity for the concept of *high need* by describing predictors useful to identify these patients [38, 41, 42, 54, 57–59]. Predictors included prior healthcare utilization, older age, number of diseases, and combination of chronic disease and biopsychosocial factors. In addition, we identified nine primary studies that provided construct, content, or predictive validity for individual predictors or the construct high utilizers [3, 5, 63–69]. Notably, a Medicare Expenditure Panel Survey study demonstrated that a definition of high needs as three or more chronic diseases and a functional limitation in ability to care for themselves or perform routine daily tasks was associated with more emergency department visits, visits with doctor or home health care, higher spending, and persistent high-cost [64]. A study using a medical complexity definition (2 + complex conditions, 6 + chronic conditions, any acute or post-acute health services utilization, indicators of frailty, and any functional impairment in activities of daily living or mobility) reported higher mortality and hospitalization rates compared to other beneficiaries [63]. Other studies documented distinct patient subgroups in latent class analyses with or without machine learning support [5, 65, 66, 69]. The evidence table (Appendix Table 4) documents the results in detail. Across all identified reviews, a recurring theme was the lack of consensus on defining and measuring high need. Issues not fully recognized in existing research sparked discussions regarding the lack of consensus on how high-need should be defined across key informants. Considerations for policy included assessing and addressing the patient’s context (e.g., food insecurity) and looking outside the healthcare system (e.g., community care providing additional resources for patients).

In preparation of the stakeholder panel meeting, panelists responded to the suggested terminology to establish a shared understanding for the population of interest. Panelists identified the term ‘high utilizers (referring to services)’ as particularly useful to describe high-need patients (mean 4.45, SD 0.66, mode 5, 55% endorsed the term as essential). All ratings are shown in Appendix Table 7 and Appendix Fig. 2 displays the number of times each term was rated as essential. Finally, when presented with 11 statements addressing the uniqueness of high-need patients, stakeholders agreed the most with the statement “*Patients’ needs are unique but there are some areas of commonality for high need patients,”* but none of the statements were rated as essential by all stakeholders (Appendix Table 8).

### Need domains relevant to the care of high-need patients

The project differentiated needs of patients, their healthcare professionals, and those of healthcare organizations caring for high-need patients.

Asking key informants about the needs of complex patients led to rich discussion about how generalizable these needs were across patients. Key informants identified social determinants that complicate care for high-need patients (e.g., homelessness) and noted a tendency to medicalize social or basic human needs (e.g., need for food and shelter) in healthcare. Furthermore, informants expressed a need for culturally competent tools (e.g., for patient engagement and health literacy) in different languages and noted that existing tools supporting clinical practice are often not specific to complex patients. The quotes in Appendix Table 2 provide insights into concrete needs expressed by personnel caring for high-need patients. The quotes highlight patient needs and the struggles that high-need patients experience in navigating a complex care environment, in addition to medical complexity.

Of the identified research syntheses identified in preparation of the stakeholder panel, only seven addressed the needs of patients [24, 29, 31, 39, 47, 49, 60]. Themes identified across studies were dealing with the physical and emotional impact of multi-morbidity, the importance of self-care, time needed to arrange medical appointments, and information needs regarding side effects and interaction of medications or conflicting advice across different conditions. Three syntheses addressed healthcare professional needs, mainly focusing on care coordination issues, such as shared care across specialists and primary care physicians, and fragmentation of care [32, 50, 60].

Our searches also identified 19 primary research publications assessing the needs of complex patients, their healthcare professionals, or both (Appendix Table 5) [70–88]. Half of these addressed patients and or healthcare professionals in the US; other countries included Canada, Sweden, Australia, and UK, and three studies were conducted across multiple countries. Most studies conducted interviews and asked about healthcare needs of high-need patients and their healthcare professionals and some studies were large-scale surveys. Of these, 12 publications referred to chronic or longstanding conditions, [70, 72, 73, 75, 76, 78, 80–84, 86] eight to multiple medical conditions, [70, 74–76, 82, 83, 86, 87] five to healthcare use, [72, 77, 79, 80, 84] four to frailty or functional limitations, [70, 82, 83, 86] and three to costs. [72, 85, 88] The evidence table in the appendix documents the elicited patient needs documented in the literature. Across 14 identified studies, responses ranged from concrete needs such as the need for simpler prescription refills, to a general need for better coordinated care [70, 72, 73, 75–77, 79, 80, 82–86, 88]. Seven studies reported on healthcare professional needs, such as the need for more time with patients and communication with other healthcare professionals for care coordination [71, 74, 75, 78, 80, 81, 87].

Needs of patients, healthcare professionals, and organizations, informed by the literature findings, were a major focus of the stakeholder panel. Panelists had rated needs identified in the literature individually in preparation of the panel meeting. The stakeholder panel discussions focused on disagreements identified in the pre-panel survey and newly nominated need domains suggested by individual panelists in the pre-panel survey. Rating results for all items are shown in Appendix Table 9. All domains confirmed as important or essential in the post-panel survey are shown in Fig. 1 as bolded.

Panelists identified 11 distinct aspects relevant to patient needs. The highest rated domain was *social circumstances*, such as housing instability. Other domains identified as essential were the *interpersonal* domain (e.g., whether the patient is supported by family members), *medical complexity*, *organizational navigation* (e.g., knowledge where to get help), *cognitive* (e.g., understanding the care sequence steps), *functional* (e.g., missing important appointments), *behavioral* (e.g., following recommendations) health, *care access*, and *trust* in healthcare professionals and the care organization. Additional important areas included *psychiatric complexity* and p*sychosocial* aspects (e.g., successful interactions with healthcare professionals and direct environment).

With respect to needs related to healthcare professionals, panelists identified *communication* (e.g., healthcare professionals engaging other healthcare professionals to coordinate care), an *interdisciplinary team* to provide care, *time* needed for high-need patients, and professional *skills* (e.g., being able to address the care complexity) as essential. In addition, professional *knowledge* about care and the system (e.g., being familiar with resources and personnel patients should be connected with) was rated as important across all stakeholders.

The highest rated domains for organizations caring for high-need patients included *staffing arrangements* (e.g., care in multidisciplinary teams), *collaboration* within and outside the healthcare system, and the *care approach and culture* in the organization (e.g., whether the organization takes a holistic care approach). Other domains judged as important were *technology* (e.g., information technology support), *finances* (supporting care for high-need patients), *investment in the workforce* such as training in complex patient care, existing *expertise* in the organization (e.g., social worker is part of the team or easily accessible to the care team), and *measurement* capabilities (e.g., being able to identify and flag high-need patients).

### Interventions for high-need patients

Results regarding interventions focus on intervention types and unintended consequences.

#### Taxonomy of interventions

Key informants disagreed on whether interventions for high-need patients are disease-specific or implementable across conditions. Key informants suggested that in practice, interventions may need to be tailored to individuals to address the specific and unique needs of individual patients. Furthermore, informants indicated that coordination between emergency and other departments and between social and healthcare organizations is critical to address patients’ needs due to a strong interdependence (e.g., a local housing initiative may empty the emergency room with emergency departments functioning as cities’ shelter systems or modern almshouses [89]).

Half (23/46) of the research syntheses identified in preparation of the panel summarized interventions for high-need patients. While some described specific interventions (mobile health apps, self-management, case management, care coordination, shared decision making, health education), others targeted broader approaches, such as the chronic care model or interdisciplinary care approaches [17–23, 27, 29, 30, 33, 36, 37, 43, 46, 48, 49, 51–53, 55, 56, 60] We also identified publications providing a taxonomy of interventions aimed at high-need patients, i.e., proposing systems to differentiate intervention types (see Appendix Table 6) [18, 20, 23, 27, 33, 36, 46, 51, 53, 88, 90–93] Taxonomies were often based on a literature review, but publications varied in scope and categorization approaches (e.g., focusing on distinct aspects such as patient goals, characteristics of successful programs, or intervention components across multi-faceted interventions). A comprehensive review identified 15 interventions, including interdisciplinary primary care, models that supplement primary care, transitional care approaches, models of acute care in patients’ homes, nurse-physician teams for residents of nursing homes, and models of comprehensive care in hospitals [20].

**Fig. 2:**
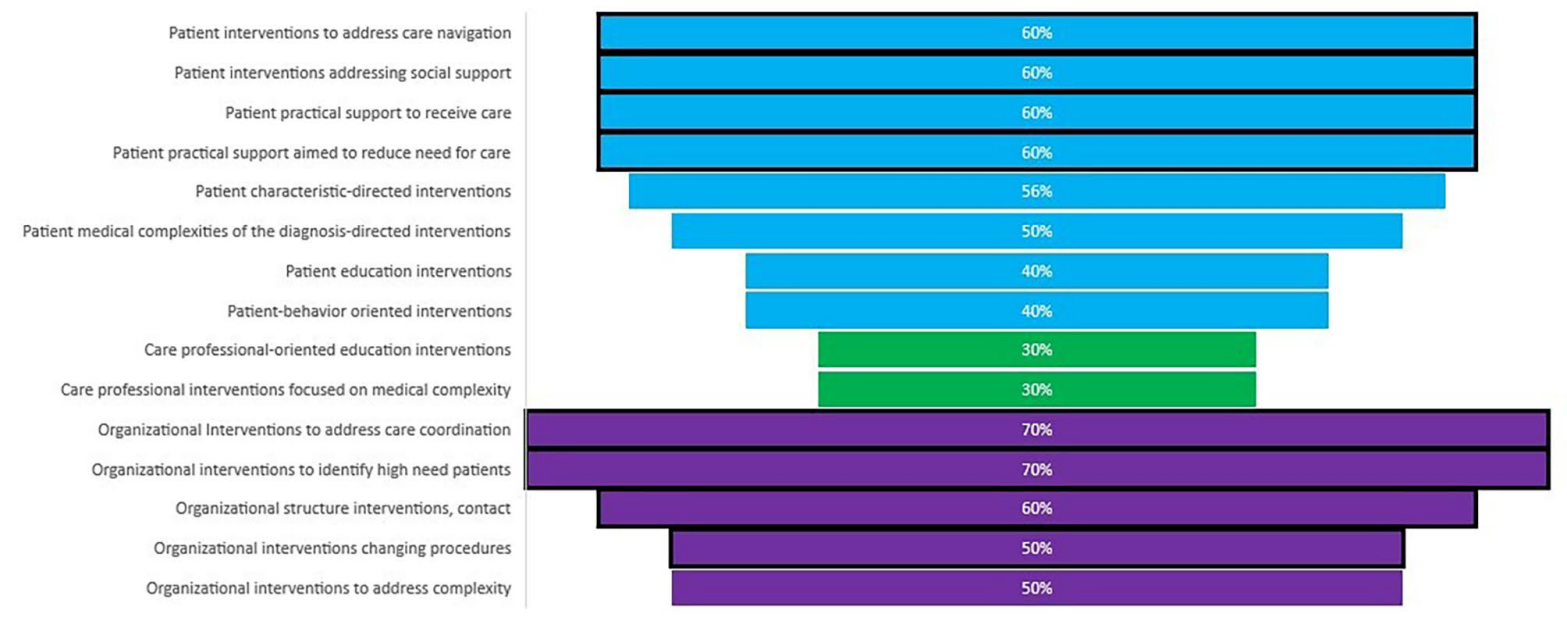
Intervention Categories Note: Size of the bar is proportionate to the number of times chosen as essential, a black frame indicates a mean rating of essential

When presented with interventions identified in the environmental scan, stakeholders endorsed 15 suggested categories as either essential or as important for a taxonomy, as depicted in the tree diagram in Fig. 2. The 15 intervention categories include interventions directed primarily at the patient, with interventions addressing social support of patients, practical support necessary to receive care (e.g., transportation), interventions potentially reducing the need for care (e.g., food bank), and care navigation rated as essential. Intervention at the healthcare professional level classified as *important* are also shown in Fig. 2; none was endorsed as essential across panelists. Organizational interventions rated as essential were processes for identification and tracking of high-need patients, care coordination strategies, changing procedures to accommodate for high-need patients, and structural changes such as implementing protocols for frequent contact scheduling and monitoring.

#### Unintended consequences of interventions

As part of the environmental scan, the key informants highlighted potential unintended consequences for patients (e.g., the need to “game the system”) and healthcare professionals (e.g., coordinating care is an additional task for healthcare professionals that are already stretched to their limits) that they had encountered in their own work with high-need patients.

Identifying unintended consequences of organizational interventions is critical to fully understanding the impacts of interventions, but none of the research syntheses identified in the literature review described unintended or negative effects of the evaluated interventions.

The stakeholder panel resulted in rich discussions about unintended consequences of interventions implemented in healthcare organizations. The pre-panel survey had elicited several potential consequences of interventions for patients, healthcare professionals, and healthcare organizations proposed by individual panelists that were discussed in detail at the panel meeting. Table 1 shows the final ratings from the post-panel survey, ordered by the central tendency magnitude.

**Table 1:** Potential unintended consequences of interventions for high-need patients

Item	Mean (SD)	Mode	Determined to be “essential”
**High costs incurred for patients**	**4.11 (1.10)**	**5**	**56%**
**Interventions may be unsustainable**	**4.00 (0.82)**	**4**	**22%**
**Screening without being able to respond to identified needs is problematic**	**3.90 (0.94)**	**4**	**10%**
**Increasing time / effort without more support**	**3.90 (0.83)**	**4**	**20%**
**Straining organizational capacity to implement interventions well**	**3.90 (0.83)**	**4**	**20%**
**Systems and insurers providing redundant interventions simultaneously**	**3.80 (1.08)**	**4**	**30%**
**New caregiver or program may fragment care and reduce continuity**	**3.80 (0.98)**	**3,4,5**	**30%**
**Increased workload, more care coordination demands**	**3.80 (0.75)**	**4**	**10%**
**Challenges with integrating new workflows for high-need patients**	**3.78 (0.92)**	**4**	**22%**
**Differences in approaches and lack of communication across services**	**3.78 (0.92)**	**4**	**22%**
**Coordination needs between programs and providers**	**3.70 (0.90)**	**4**	**20%**
**Changes in funding and leadership can affect programs**	**3.70 (0.78)**	**4**	**10%**
Backlash from providers if care is required to be more standardized	3.40 (1.28)	2	30%
Performance measures need to be redefined (e.g., incorporate patient satisfaction)	3.40 (1.28)	2	30%
Increased costs	3.40 (1.11)	4	10%
Withdrawal after resources end	3.40 (1.02)	4	10%
Compassion / empathy fatigue	3.40 (0.92)	4	10%
Patients feel labeled	3.40 (0.80)	3	10%
Resources can be overwhelming for patients	3.30 (1.42)	2,5	30%
Programs aim to reduce healthcare utilization but can inadvertently increase utilization by exposing unmet needs or intervening when something might resolve without intervention	3.30 (1.35)	3	30%
Standard rather than patient-tailored care	3.20 (1.17)	2	20%
More burden for patients (more phone calls, visits)	3.20 (0.98)	2,3,4	10%
Clashes with performance measures	3.10 (1.14)	2	10%
Patient satisfaction negatively impacted when supportive intervention ends (“what will I do now?”)	3.10 (1.14)	3,4	10%
Patients might get too attached to a program that will end	3.10 (1.04)	2	10%
Providing housing to homeless patients can cut off access to social network for support	3.10 (1.04)	3	10%
Potential mismatching of interventions with need profile	3.10 (0.83)	4	0%
Initial worsening in health service utilization outcomes	3.00 (0.89)	3	10%
Provision of unneeded services	3.00 (0.77)	3,4,5	0%
Patients gaming the system	2.90 (1.22)	4	20%
Decreased self-efficacy in patients (overreliance on provider, perception of self-efficacy suffers)	2.70 (1.27)	2	10%
Decreased autonomy perceived by patients	2.50 (0.92)	3	0%

Note: The table shows the endorsement of panelists of the relevance of potential unintended consequences. Mean, standard deviation (SD), and mode refer to a rating scale ranging from 1 (not important) to 5 (essential). The last column shows the percentage of panelists determining the unintended consequence to be considered essential. Items at the top of the table (bolded) were endorsed as important across panelists

While none of the identified possible unintended consequences were seen as an essential aspect of care, panelists identified 12 potential consequences of interventions as important (see top half of Table 1). Of these, several would affect patients directly, such as *high costs incurred for patients* (e.g., through co-payment or needed resources) and *fragmented care due to a new caregiver or program*. Others would primarily impact healthcare professionals (e.g., *increased workload, more care coordination demands*) and the healthcare system (e.g., *interventions may be unsustainable*).

## Discussion

This project comprised of an environmental scan and stakeholder panel. The environmental scan explored the concept of *high need* further with key informants and provided an overview of the published research base on high-need patients. The stakeholder panel used modified Delphi methodology to determine domains of need in this population and healthcare professionals and organizations caring for them and to determine characteristics of interventions for high-need patients, their healthcare professionals, and healthcare organizations caring for high-need patients.

Stakeholders reviewed conceptual frameworks and terminology of high need and established that individual patients’ needs are unique but all high-need patients share some common features. As documented in multiple research publications, the lack of consensus on terminology hinders research and policy [2, 5, 43, 94–96]. Panelists agreed that healthcare utilization is a key characteristic of high-need patients; however, focus on utilization and spending is problematic as intense use is often temporary [97]. In recent years, researchers have started to collate existing measures to identify high-need patients, [41, 44] to assess multi-morbidity, [25, 26, 34, 61] and to document treatment preferences and results. [40, 45, 98] In addition, recommendations for treatment of multimorbidity, [50] a collection of conceptual models of patient complexity, [62] and research agendas to support patients with multiple conditions [95, 99] have been published recently, which may advance research and policy for high-need patients more broadly.

Panelists agreed on domains describing patient, healthcare professionals, and care organization needs. The findings recognized the complexity of the identified needs and established that eleven different domains should be differentiated for patients (e.g., needs routed in social circumstances such as homelessness versus needs routed in the medical complexity of diagnoses, such as possible drug interactions resulting from polymedication). Similarly, there are five different healthcare professional needs that should be addressed through interventions and resource support (e.g., communication needs and skills versus having access to an interdisciplinary team). Organizational need domains are also diverse and offer multiple entry points for organizational changes (e.g., the need to make staffing arrangements including adjusted panel sizes versus the need for collaboration within and outside the healthcare system) as indicated in the eight identified domains. The diversity in needs highlights the necessity to explore areas beyond the simple operationalization of counting the number of chronic conditions to describe high-need patients or defining patient complexity as morbidity alone [62, 96]. It highlights the multi-level and multi-component approach that is needed to care for high-need patients.

Our taxonomy of interventions was informed by published intervention categorizations as well as panel discussions. The taxonomy includes 15 types of interventions and reflects the diversity of approaches to support high-need patients. Not all interventions are directed at patients (e.g., providing practical support promoting access to care), as some approaches may also target healthcare professionals (e.g., changing clinician behavior) or implement organizational changes (e.g., introducing mechanisms to identify high-need patients). Our literature review identified over 20 published evidence syntheses summarizing the effects of interventions for high-need patients–all providing information on different interventions, components, or approaches–highlighting the diversity of this field. In addition to and independently from patients, healthcare professionals also benefit from support, and structural interventions can benefit patients, professionals, and organizations [100–106].

Our project also addressed potential unintended consequences of interventions for high-need patients. The panelists agreed that the top unintended consequences to consider include costs incurred for patients, unsustainable interventions, screening for needs without resources to respond appropriately, increased time and effort for healthcare professionals, and straining organizational capacity. Some of the unintended consequences may be mitigated with careful planning or may be avoided altogether, for example, through effective care coordination. However, our study shows that interventions should be addressed from a systems approach that measures effects from different perspectives, including patients, healthcare professionals, and organizations. In addition, many interventions for high-need patients are complex, with multi-components, which in itself poses evaluation challenges, reinforcing the need for careful measurement of effects [27].

Our study has several strengths and weaknesses. We identified a large number of relevant scientific publications and explored the concept of high need with key informants to thoroughly prepare for the stakeholder panel. We followed established methodology and incorporated diverse perspectives from relevant stakeholders. However, it should be noted that we used rapid review methodology rather than conducting full systematic reviews for the topics of interest, and thus may have missed relevant publications. Despite a framework-guided approach, the panelists are selected representatives of stakeholder groups and do not necessarily cover the universe of opinions. The limited number of panelists enabled meaningful group discussions and consensus finding, consistent with the RAND/UCLA Appropriateness Method and allowed elicitation of diverse opinions while also allowing consensus to emerge [14, 16]. It should be noted that the panel did not include family or other non-professional care givers; future research should pay particular attention to this important group. In addition, multiple panelists had a VA Health System affiliation and future research should determine whether and which needs do not generalize to other healthcare systems that care for high-need patients.

We demonstrated, in a multi-faceted, evidence-based and expert-informed research project, that care for high-need patients requires a thoughtful approach. Differentiating need domains provides multiple entry points for interventions directed at patients, healthcare professionals, and organizations. Intervention evaluations should consider the intended and unintended effects downstream effects on all stakeholders; most importantly high-need patients, their healthcare professionals, and the healthcare organization caring for this population.

### Electronic supplementary material

Below is the link to the electronic supplementary material.

Supplementary Material 1

## Data Availability

All data generated or analyzed during this study are included in this manuscript and its supplementary information files in de-identified format.
