# Supplementary material for: Caring for high-need patients
Source: BMC Health Serv Res. 2023 Nov 23;23:1289. doi: 10.1186/s12913-023-10236-w (PMC10668484; doi:10.1186/s12913-023-10236-w)
Supplement: Supplementary file 1 — Supplementary Material 1 [file 12913_2023_10236_MOESM1_ESM.docx]

# **Online Appendix**

**Contents**

[Online Appendix 1](#_Toc128323001)

[Appendix Table 1: Key Informant Interviews 2](#_Toc128323002)

[Appendix Table 2: Quotes from Patient Aligned Care Team Intensive Management Project Healthcare Professionals Interviews 9](#_Toc128323003)

[Literature Review Search Strategies 16](#_Toc128323004)

[Literature Review Eligibility Criteria 17](#_Toc128323005)

[Appendix Figure 1: Literature Review Flow Diagram 18](#_Toc128323006)

[Appendix Table 3: Literature Review Evidence Table Research Syntheses 19](#_Toc128323007)

[Appendix Table 4: Literature Review Evidence Table Validity Studies 40](#_Toc128323008)

[Appendix Table 5: Literature Review Evidence Table Needs Assessments 42](#_Toc128323009)

[Appendix Table 6: Literature Review Evidence Table Intervention Taxonomy 46](#_Toc128323010)

[Panel Composition and Stakeholder Representation 49](#_Toc128323011)

[Appendix Table 7: Stakeholder Endorsement of Terminology 50](#_Toc128323012)

[Appendix Table 8: Stakeholder Endorsement of Statements Uniqueness vs Communalities 51](#_Toc128323013)

[Appendix Figure 2: Need of High-Needs Patients, Their Care Professionals, and Care Organizations 52](#_Toc128323014)

[Appendix Table 9: Stakeholder Endorsement of Needs 53](#_Toc128323015)

[Appendix Table 10: Stakeholder Endorsement of Intervention Categories 54](#_Toc128323016)

[References 55](#_Toc128323017)

## **Appendix Table 1: Key Informant Interviews**

| **Key Informant**  **Work related to high-need patients** | **How is high-need or complex defined?** | **What are the needs of complex patients?** | **What are the needs of primary care providers?** | **What interventions have been suggested to meet the need of complex patients?** | **Are there unintended consequences of interventions to address high-need patients?** | **Are there issues not fully recognized in existing research or policy making** | **Which tools are available to support complex patients, their healthcare providers, and healthcare delivery organizations?** |
| --- | --- | --- | --- | --- | --- | --- | --- |
| High need literature synthesis expert  I'm leading this review called the “Management of High-Need, High-Cost Patients”. It is a realist and systematic review of intervention and exposure questions in terms of identifying high need, high-cost patients. The realist review portion is trying to understand the mechanisms that affect health care use and improved outcome. | This is a complex area to work in. One of the issues is whether a person has had the overuse or inappropriate use of services. How do we predict how somebody will need particular things? We ended up talking about the overuse of emergency room or inpatient services. That’s an area that Camden RCT came out with trying to identify. We’re focusing on potentially preventable or modifiable high use of healthcare services. People are somehow using services that the system considers to be inappropriate that's high use of emergency room care. If individuals have cancer or some other particular need, those are the kind of needs where you think that they could be taken care of in a PCP kind of environment. The first question is identified as populations. Who are these people? We're looking at prediction models. We're looking at multivariate regression in terms of how people are identifying them. There's a lot of literature out there. Is it the social determinants of health? The goal is for it to be actionable. Our first task is to go through the literature and figure out how to identify a population. We’re looking for quantitative studies and qualitative research to understand the populations. We are working on a definition, but we have not finalized a definition. The issues have to do with how long somebody has used these kinds of services in the group. | We are still doing our literature review and haven't summarized it yet. We don't have a final answer on that. When you are talking about needs, we are not looking at the fine grain you are looking for. We are looking at a lot on care-management because that seems to be at the patient level. The needs are different based upon the population. If you’re talking about the frail elderly and the people who have issues of isolation, their needs and interventions may be different. Do young adult populations have chronic condition needs they’re not managing well enough? Based on particular patient needs and social determinants of care (i.e. living in a rural area, access to care, etc.) will determine the kind of intervention. We had our key informant group. We found a split between those that said these interventions must be disease-specific and those that said they needed something that could be implemented across disease conditions. There’s no consensus yet. | I don't have any answers for that yet. It depends on how you want to define "interventions". I have some background in doing health literacy interviews and interventions; there’s a lot of literature on that. We’re also looking at CMS interventions. We’re looking at all the different levels. What is the system doing? What incentive is the system creating in order for the clinician to change the way they practice? Cultural competency and language (i.e. translators) are other areas as well. | There’s nothing that I could recommend at this point. In terms of interventions, we haven't found a lot of things because we have required a comparison group. There might a lot more out there if you are broader and do pre-post studies. I think it is way too strong to say that you are going to stick only with RCTs. I haven't read the CAMDEN study in detail but it’s only one study. The CAMDEN study had a relatively not stringent inclusion criterion and that is what happened with us. We had to have twelve months worth of data of high use in order to be included in our intervention studies. The CAMDEN study had 6 months. We are re-evaluating our inclusion criteria to see if we missed any studies that have 6 months. If they had a more stringent inclusion criterion, they might have had different results. You should look across studies. | I don't have any answers for you. We are keeping this in mind as we are looking at CMS interventions. Do alternative payment models create an environment that makes it more doable? That creates a situation where clinicians are more able to do these additional activities, rather than having it be one more thing they have to do that they’re not being compensated for. | One of the things that are overlooked is looking at a series of how things are intended to work and how they are actually working. Starting from a perspective on what they are trying to find and what might their quantitative results show them. That is what I am looking at in my research. Regarding policymaking, the field doesn’t have a good answer. If you're looking for specific characteristics that somehow make up social determinants of health concern, there’s no agreement on that. How do you actually weigh different things relative to another? Also, I’ve been thinking about what’s actionable. What can people actually do with the information that you’re finding? If I tell you that women tend to be more high need and high cost than men, that wouldn’t be actionable from a policy perspective. Try to keep what’s actionable in the front of your mind. The answers that are coming out around CAMDEN is what are the alternatives in the system. Even if we come up with this amazing, preventable and modifiable definition, it’s still within the context of the healthcare system that they have access to. They have to have a PCP that they can see and feel that they can communicate with. It’s a complicated scenario without just one answer.  Perspective matters a great deal. Some things are modifiable though it may not be in different situations. You have to think about the perspective of whether this is something a health system is trying to implement. Is this modifiable based on the entity vs. the individual? | There is the health literacy tool that we created at RTI. Our review is not focused on that. |
| ER physician  Work with high need patients - this is mostly what we do with the county. I am an ER physician who works at Los Angeles County+USC Medical Center which is the largest safety-net hospital in LA County. It is adjacent to Skid Row and areas that have high-needs patients. | The term in the County setting is "needs special assistance". You’re talking about multi-diagnoses folks that are often suffering from a range of social, medical, mental health, and/or substance use problems. | I think that the main needs are treatment for social determinants of health being your typical housing, food insufficiency, financial insolvency, and all of these issues that flag our patients and we over medicalize them. One of the patients we had was a 19-year-old man that is homeless who has a possible diagnosis of bipolar and a history of mental health issues. He also was not eating. His substance use was because he was living off the streets which makes it hard to sleep and cope with anxiety. He had a triple diagnosis and he is labeled as someone who is suffering from mental health problems. He was refusing to eat which seemed to be a coping mechanism. He was anorexic and he had all these abnormalities as a result. He was so upset about the fact that he’s homeless right now but his label in the chart shows substance use and mental health, rather than the real issue of him using maladaptive coping mechanisms for being homeless and not being able to take care of a lot of his general needs. | For the most part, it’s things that allow us to move more upstream. The majority of our armamentarium in the emergency department is so downstream. The best thing I can do for somebody is a site consult to get them housed for three days but I can't do anything about the fact that they've been coming in and out of the ED. With the questionable suicidality that sometimes does or doesn’t get them housed for that three-day stay, you’re looking at all these things where we can order the utmost of medication to correct things and to try to treat their medical problems, but we can't do the normal things like give them stable food or basic necessities of life. It’s often presented to us as medical problems. It’s not uncommon that when you talk to this individual about his back pain, he’s actually just hoping to nap for three hours because it’s the middle of the night and he's unsafe because he lives in a tent in a dangerous area and he’s heard that his neighbor got attacked. | There were several housing initiatives that used to be more common. There were big pushes for housing for health. Sometimes, we wouldn’t see patients for a while and it was because they got housed. There were some of those interventions. We tried to do a lot with the wellness center, specifically to get people enrolled in primary care and get them medical homes as well as insurance and legal aid. Those interventions have been helpful. When the housing push first came in, a lot of the people we saw on a daily basis were gone. If they were to lose their housing, then we would see them again. | At the root of this is moral hazard. Some of it plays into the adaptive behaviors we see in many of our high utilizers or our special needs patients. They know how to gain the system a little bit and they know that they can get someplace to stay if they say they are suicidal. They have a plan and they might say they’re going to jump off a bridge. Many people do commit suicide that way. There’s moral hazard in the fact that we give sack lunches and food in the ER. My problem is not that we give food and people come in for it. It’s that we can’t be as overt as we should be about it. Sometimes people have things that they really need but it’s not something that the emergency room is willingly giving. | Everyone is obsessed with return investment. I recently saw the paper about the CAMDEN project and we are trying to do a couple of small interventions in the health care system. There could be a return investment if you tried a full-scale social needs treatment arm on these high needs patients and invest heavily in housing and more specific interventions and looked well enough out to see changes in spending and efficiency. I don’t know why we in the ER can't figure out a successful food diversion plan where we put food pantry access in the waiting room. It’s reasonable from the standpoint of the patient to utilize these services. | There are some technological programs that are promising. The real devil is in the intervention arm at the other side. I think it’s interesting what Healthify has done and some of these online platforms to treat social needs. I know that a lot of people are using a social service chatbox. Often on the calls with DHS and groups that are trying to do a lot of these screenings are incorporating this social determinant screen. The latest one was the opioid risk. The problem is that there are only certain things that we have interventions for. Most of the time when you are screening for these difficult problems from the medical standpoint, we often don't have a good intervention arm on the other side of it. |
| Social worker perspective  When I worked on the PIM project, the team was already established and I came on as the first social worker. We tried to identify patients that had more case management needs. I would put them as complex high need patients that needed more assistance who couldn't do or reach things on their own. There were patients who had different barriers such as psychosocial barriers to care. One of them was linking patients with resources. We initiated taking patients to places of care and signing them up for public benefits (i.e. financial assistance, food vouchers, etc.). Sometimes those barriers would make them high needs because psychosocial needs weren't addressed. | It’s individualized. For example, one person might have issues with finances because they might not have enough income and they just need to be linked to benefits. It could be because they have poor financial literacy and can't manage their income. It could be because they have addictions and deplete their income to feed their addictions. It could be because their memory is declining and they need someone to manage their affairs. It could be coming from different sources. It needs individualized attention and the time to figure out the source of the problem so that it’s not generalized as one issue. | From a patient standpoint, it’s very useful and makes a big difference to have someone available so that they don't have to be transferred multiple times to reach one person. Having immediate access to one person without having to pass them along makes a big difference especially for a big healthcare system like the VA. Patients want to access different resources but the consulting team needs to be responsive and call the patients as patients get lost. They benefit from someone guiding them through the processes without interruption. I have seen that this needs to be improved for programs to be more connected. On the provider end, when you know that you have a patient that is interested in any kind of service, it’s helpful for the follow through and follow up to be more streamlined. That is where my job was important; I was helping patients connect the dots so that they don't get lost in the process. I would help them navigate through it. It helps to have one point of contact that can also be there when the patient has a question. That way, the patient has an advocate in their corner so that they don't give up on it.  How do you prioritize?  The key word is prioritizing. When patients came to see me as a social worker, I had so many patients so it may be addressing one thing with the patient and calling their family to get them more support through informal channels. If there was an issue with transportation, we could help them apply for public transportation. However, they’d still need someone to call transportation when they need it and remind them about the appointment. They would really need family support. If there wasn’t family support, it would be a question of whether or not they could live on their own. I have learned a lot about networking and linking patients with programs at any given time that may have more ability to stay with them longer. We got a call from a patient’s neighbor who was very concerned. The patient was failing at living on his own and there were concerns about his age, health and lack of support. When I was called as a PACT social worker, I spent time talking to the patient and the neighbor trying to prioritize. We made a referral to the home base team to do a home assessment, and then there was a referral we made to a mental health program that was focused on case management. We started with that and then we connected him with the resources we had available. We don't always have the time with such a big caseload. Sometimes it’s more than the office and hospital setting. Sometimes it’s about what is happening on the outside and with the patient at home. We try to prioritize and bring in more team members (caregiver or refer out to other specialties) to help. It’s also something I try to do when I am with the patient and trying to make that connection when they are in the office. Work with them in the community.  How insightful are patients about their own needs?  It has to do with their own health literacy and literacy in general, their background, their personal values and what is important to them. As their social worker, I might be recognizing that things that are recommended by the therapist and physicians, and they could be coming from a different belief system. See patients from a whole perspective and be culturally sensitive for patients having different beliefs than the traditional healthcare setting. | Follow through is not transparent for the patient. | Personally, the most important thing to address is what the patient identifies as important to them. Even though they might be chronically ill with many different things going on, a lot of times its just one issue that is really persistent and more urgent to them. Focusing on what’s important to them helps with establishing a connection with the patient and allows them to develop trust and be able to see their healthcare team as somebody they can turn to. One challenge is to step back from interventions and recommendations and what we think that patients would benefit from. It’s important to honor the patient’s choice even if those choices may be detrimental to their health. They have the right to make those choices and it’s important to be there for them and honor their decisions. | It’s about the timing. I can think of many different times when patients weren't ready for something at that point in time. When I was in the ER, I had a patient that was in a domestic violence relationship and we would have conversations about safety and well-being. There were multiple ER visits and one day the patient called and said she left the partner that was abusing her. Sometimes you have to sit with the patient and give them the education, tools, and time to listen to them and let them live their lives. We are there but we can't make them live a life that we want. It’s their life. | I don't know if I have necessarily thought about research. Sometimes I feel that people get stuck in their own point of view instead of looking at the bigger picture. Sometimes when a patient comes to us with an issue, we automatically look on the inside at the resources that we have and we overlook what we can provide to the patient (i.e. community resources) outside of the VA that can improve their quality of life. They have the choice to choose the care that they want. I don’t know how much this has been studied, but I would be curious if there is research about the connection between external partnerships and the value they have on existing gaps in care | My biggest surprise when I left PIM and worked on a PACT team was that I didn't appreciate the fact that our primary care team has such a huge caseload. When there is such a big caseload, the interventions are going to be different and more time-sensitive. Solutions will be more immediate and tailored to time. Some problems may be solved that way, some may not be, and that is okay. You can give the patients the tools and it might be a period of time that they won't use it and you have to tailor it to each individual patient. Do your best to be there for them and not expect that there will be an immediate outcome. I don't know if there’s any particular tools other than a 20-minute appointment with a patient and linking them with the right resource or person that could be their point of contact so that they could have a connection. Making a personal relationship is the most effective tool there can be. Take the time to get people the basic things that they need help with getting for whatever reason or barrier they may be experiencing. |

Notes: CMS Centers for Medicare & Medicaid Services, PACT Patient Aligned Care Team, PCP primary care practice, PIM PACT Intensive Management

## **Appendix Table 2: Quotes from Patient Aligned Care Team Intensive Management Project Healthcare Professionals Interviews**

| **High-risk patient struggle or need** | **Quote** |
| --- | --- |
| Social determinants of health | “…our medic found out that one of the patients wanted to leave the hospital because he had to pay his bills. He was worried they were going to take away his furniture if he didn’t go pay his bill, so he was able to figure out what the problem was, and he had to help take the guy to go pay his bill and bring him back, so you have to look at the whole patient.”  “We have one patient who has…high blood sugar…I mean, he’s very sick because of this. But…He’s worried about being evicted from his home. So, in his mind, getting his home situated is more important than addressing his blood sugars, and we understand this. So, in our IDT meeting, that’s more my area [as a social worker], so I will work with the Veteran to help him or her become stable, so they can engage more into their healthcare.”  “…we look at them [the PIM patient] as a whole and what is taking place in their life right now that’s affecting their health besides just the medical…For instance, you have somebody that’s lost a spouse. Okay. The spouse did all the cooking for them at the time to help them keep their diabetes under control. The spouse passes away, now, all of a sudden, you’ve got a gentleman that now does not cook for himself. So, he buys things that go in the microwave that have to be cooked a certain way. He’s not on that same diet or menu plan. So now what’s taking place is he’s not eating correctly and now his diabetes is out of control.”  “…Your average normal patient that comes in with diabetes you’re going to want them to have a hemoglobin A1C goal. These patients, that’s not feasible. Sometimes these patients, their biggest thing is housing, or their biggest thing is their fish, getting their fish tank taken care of before they’ll even take their insulin. A regular primary care role, they don’t have the staff or the time to be able to take a look at these patients to see how many times they’ve been in emergency room, what services they need, what worked in the past, what doesn’t work, to use harm reduction, to strategize, to see what didn’t work and what can work.” [PACT vs PIM, chronic conditions, whole health and psycho-social needs]  And [one patient] he’s just like I’m not that interested in talking about my substance abuse. I really want PTSD treatment. And we sort of talked to him, but he’s like, but I want to keep seeing them [PTSD MH providers] because I trust them. And so we sort of like helped make that connection. And they’re like, well, we’d love to see him, but he doesn’t have transportation and so he’s very sporadic in his visits. So, our team got him transportation based on his non-mental health issues. He has this chronic pain, he finally saw ortho clinic because he had transportation and found out that he needs total hip replacement. And they told him that they’re not going to do it unless he has clean utoxes for four months. And so, all of a sudden he’s now interested in substance abuse treatment.” [transportation = social determinant of health component; see also substance use & mental health below]  “[A patient] who has a history of, like, traumatic childhood, never really having a stable home, no support system, used to live independently, was evicted from his housing, has recurrent foot fungus…he was supposed to go to the downtown clinic every day to get wrapped because of the genesis of it, and he wasn’t going and so part of that was really just listening to the Vet. He was very wary of mental health and didn’t want to take any psych meds and didn’t want anyone in his home, but then, as we kind of built that rapport, that trust, he was able to build the most rapport with me, so I became kind of like the primary case manager and so I was like, ‘Well, why don’t we walk downstairs to the downtown clinic?’ And slowly but surely he was able to go on his own.” [Also fits with “relationship building, trust”] |
| Substance use disorder, with or without co-occurring Mental Health Issues | “…When we dig a little deeper, it turns out that the reason that the PACT teamlets just feel like there’s no goals [regarding some high need patients] is because they’ve just kind of given up, some of the severe alcoholics and some of the severely noncompliant patients. And we’ve actually had some of the biggest impact on some of those: On helping to keep people dry, helping with identifying why it is the patient is so non-adherent.” [SUD; Providers talking about PACT vs PIM]  “[One patient has been] one of those that’s a difficult one because he’s got a lot of anxiety, had a lot of substance abuse history, just to get him involved in his own care. And he’s also a fairly dependent personality so trying to move him towards the place of making his own appointments, cancelling his own appointments when he can't go, and doing those sorts of things. But we started at a place where he was kind of like resistant in some ways but built trust with him by helping him when he needed it and how he needed it. And it like, again, got him into a stable living environment. He was in a very unstable living environment.” [mental health issues; substance use; building trust/relationships]  And [one patient] he’s just like I’m not that interested in talking about my substance abuse. I really want PTSD treatment. And we sort of talked to him, but he’s like, but I want to keep seeing them [PTSD mental health providers] because I trust them. And so we sort of like helped make that connection. ….He has this chronic pain, he finally saw ortho clinic because he had transportation and found out that he needs total hip replacement. And they told him that they’re not going to do it unless he has clean utoxes for four months. And so, all of a sudden he’s now interested in substance abuse treatment.” [Also fits with relationships and multimorbidity and social determinants of health]  “So I’ll give you an example of someone who has polysubstance abuse, depression, PTSD, diabetes, hypertension, obesity, heart disease. And that would not be an atypical patient for us. He’s in his 50s, believe it or not. And he is involved in therapy and he goes to groups, that sort of thing. But after coordinating with all of his treating providers, I kind of came to the conclusion that he was just kind of not really fully engaged and maybe not being totally upfront about his substance use and he was also very, very depressed and would miss kind of important appointments. …. So I worked with him for probably two or three different sessions of psychoeducation, motivational interviewing, and he did eventually start on an antidepressant prescribed by our psychiatrist and has been doing a lot better.” [also included under managing chronic conditions]  “One particular patient I have, he has cancer, newly diagnosed multiple myeloma and he’s also a recovering addict and has had a few setbacks or relapses with his drug of choice and during those times he may have strayed away a little bit from his weekly chemotherapy and radiation, and so myself, as well as at the time the first co-visit team visit, the social worker came with this particular patient, as well as some of his siblings that are caring for him and concerned about his care, as well as we met with the oncologist and the nurse practitioner oncologist.” |
| Mental health | “…this population has a lot more complex trauma, especially sexual trauma, so I am very careful about how I think about interacting with them. I probably spend more time building rapport…than usual as opposed to just kind of diving right in and getting information that you need and sending them along. I use my motivational interviewing skills a lot, probably with every single interaction. Yeah, probably a softer approach; probably a slower approach because it’s just, in general, kind of a more sensitive population.”  Q: …is there anything that you would consider like successes so far or what the greatest success of the program has been?  A: Oh, yeah. I think that gentleman…he’s a Vietnam Era Vet…and then he’s been around the VA since the ‘70s and well known by many of the nurses on staff…this particular Vet, who we got his prosthetic limb, his leg, out of storage, was very challenging because this individual is very kind of personality-driven and changes his mind, one day he’s up, one day he’s down. But getting that leg for him and getting him back into mental health to where he’s going regularly…I feel like that particular patient, he’s not dancing on the moon but, boy, I think we’ve made a lot of progress with him. And for years, for decades, he has just kind of been—I’ve heard all the stories, he’s been banned from certain parts of the hospital because of his behavior. He’s been banned from the clinic I’m standing in because of his behavior. So the fact that we were able to get him engaged with mental health, with the stuff he needed, yeah, that was a definite win.” [See quote about same Vet in chronic condition management]  Often times- it’s a big organization, the VA; very complex, very tough to navigate, even if you have a sane, straight, healthy mind. So you can imagine for some of our Veterans who have PTSD, depression, anxiety, some type of psychotic disorder, it’s very difficult just to navigate the world let alone the VA system which can be admittedly sometimes very convoluted. So I really enjoy the fact that our team and my position as a nurse, I can talk one on one with the patients and really find out what's going on. Maybe there's a psychosis thing. Maybe they need to reengage in mental health. Maybe it’s a misunderstanding. Maybe it’s anxiety, PTSD, which we see a lot of; the hallmarks of are avoidance. So you see someone not going to appointments, it may be because they're too anxious to walk into the building. So, we really have to keep a very open mind and understand that everyone we see here is to be helped in some way, shape, or form—and generally can be helped.”  “She [patient’s wife] was like, “He’s like a whole new man.” This particular case, they’ve been married for 27 years and she said he’s never talked about his trauma in Vietnam but after [PIM] getting him with mental health psychiatrist, after having an outlet, he actually sat down with her one night and told her his whole story and she just typed it as she read and she said she’d never known these things, and she had been married to the guy for 27 years.” |
| Physical health | “’I want to get some of this weight off of me.’ That’s one of the biggest things we hear. ‘Okay, well, how can you get your weight improved? Part of it will be changing your diet. Part of it will be being compliant with your medications. Part of will be possibly doing some physical activity’, so that’s how we identify for those Veterans, so sometimes they have their own, sometimes we’re able to collaborate together and sometimes we will suggest them.” [This could also be in “chronic condition management” and/or “lifestyle change”]  “I went in the home and [a patient] was really struggling with his bed and getting in and out because he has one-sided weakness. And so we talked and…I got him a hospital bed and then he’s very happy with that. Been following up—initially he was like, no, I’m fine, I don’t think I need you. Well, then he had a fall, then he came back to the hospital, came to the hospital and then when he went home I went back in and we started talking about more issues and now both him and his niece are calling me because I told them, I said, you know, you’re prone to urinary tract infections; these are the things you need to watch for. So they call me, I feel like this, this is what’s happening. What do you think I should do?” |
| Multimorbidity or medical complexity | “I’ve had to switch in to my mental health role a couple of time. I had a veteran who was going in to prostate surgery and was really dealing with anxiety about that, almost about to pull out of going to surgery. So, we did some work on the anxiety that he was having around that. So, you have to be flexible with what comes in the door and using whatever works that day with the ultimate vision being where they’re wanting to be.”  “…we’ve been working with the Veteran for six months and basically what happened was he hoards his medications in the closet and hides them from everybody and we’ve been working with him on this issue, but I was like, “You know what? I think this guy at present needs home-based primary care because like as a clinic provider you will never know what he’s actually taking unless you’re like physically in there, kind of like looking at what he puts in his medication box because he kind of switches them. And I’ve been there now like maybe like six times and every time I go there it’s different and he’s claiming he’s out of things. And I was just like this is a long-term issue which is going to be really difficult for a clinic provider to be able to resolve. You’ll never know what medications the patient’s on. And so, I recommend that he go to home-based primary care.” And the primary care provider would be like, “Oh, that sounds great. I would have never thought that he would qualify for home-based primary care because he can still come to clinic.” I’m like, “Yes, he can still come to clinic but, you know, clinic isn’t providing him optimal care, and so that’s acceptable for home-based primary care.””  “There’s a gentleman that we see. This particular Veteran has a progressive neurologic disorder and the primary care physician…said, “Hey, this individual’s having issues with eating, nutrition, and apparently he doesn't look very well put together, you need to see the condition of his house because he’s going to be subject to weakness and falling. And we have to make sure his environment is modified in such a way that it’ll be safe for him.” …So…the place was a mess. Food was left out… He was hoarding. …You could hear mice or something running around in the papers and junk he had all in his room. So, you get to see the picture of what the provider doesn’t see; they kind of get the hint that something’s not quite right at home.”  “…this is a guy who has a history of, like, traumatic childhood, never really having a stable home, no support system, used to live independently, was evicted from his housing, has recurrent foot fungus that he has to go—he was supposed to go to the downtown clinic every day to get wrapped…and he wasn’t going and so part of that was really just listening to the Vet. He was very wary of mental health and didn’t want to take any psych meds and didn’t want anyone in his home, but then, as we kind of built that rapport, that trust, he was able to build the most rapport with me, so I became…the primary case manager and so I was like, “Well, why don’t we walk downstairs to the downtown clinic?” And, slowly but surely, he was able to go on his own. …and he actually doesn’t have to get his feet wrapped anymore, and he’s started seeing a psychiatrist, and started an antidepressant, and is less tangential and more focused on action. But I think, really, without kind of the sitting and listening and kind of focusing on his goals, although sometimes the goals were way out there, that probably wouldn’t be attained… He became more conscious of his health and agreeable to follow up regularly for his care and hasn’t been hospitalized or had any emergency room visits for about four months.”  “So, I’ll give you an example of someone who has polysubstance abuse, depression, PTSD, diabetes, hypertension, obesity, heart disease. And that would not be an atypical patient for us. He’s in his 50s, believe it or not, and he is involved in therapy and he goes to groups, that sort of thing. But, after coordinating with all of his treating providers, I kind of came to the conclusion that he was just kind of not really fully engaged and maybe not being totally upfront about his substance use, and he was also very, very depressed and would miss kind of important appointments. …So, one of the things that I was able to do for him was meet him in a clinic visit and kind of coordinate with existing providers and did several co-visits with some of his existing providers and developed trust that way. …And the team and a lot of his other providers had really determined that one of the things that would be helpful is starting him on an antidepressant, which he’d been really leery to do because he had had bad side effects in the past. But it was honestly probably drug use in the past. His A1C was kind of out of control and so starting on an antidepressant was just a great idea. So, I worked with him for probably two or three different sessions of psychoeducation, motivational interviewing, and he did eventually start on an antidepressant prescribed by our psychiatrist and has been doing a lot better.”  Sometimes you can only maybe get to one or two people in a day because of their multiple comorbidities and the needs that they have and coordinating of services. For example, we had a patient that required new means testing. I got temporary approval for travel to get them here so I could get them in, get them seen. The patient had end stage renal disease. The patient had uncontrolled diabetes. He had vascular dementia. He had the medical foster home. He was unable to have ATS intervene because of his age. He got C. diff. We went to the home to try to ensure he was taking his meds. We had to get guardianship. So there was a lot of work involved to get this patient…I consulted Ethics, we had a whole team meeting with all the interdisciplinary team involved.” |
| Chronic Condition or Disease Management | “Personally, one of the oddest things I’ve done is go out, help a Veteran get his prosthetic leg which was in a storage locker, so he could start this goal of losing weight, getting his A1C down, because he’s diabetic. …And sure enough…we got the prosthetic for him, dug through the storage and got it for him, but he’s actually walking now. Not all the time, but he can go to physical therapy, and I think he walks 60 feet, which for this man, he’s been chair-bound for a long time because of his amputated right leg—below-the-knee amputation—well, is a miracle. So, his sugars are under control. There's still some mood issues to contend with, but physically he’s really turned the corner since we’ve been involved.”  “…a lot of these Vets can't sleep, and…maybe years ago, they got a CPAP machine but they hated it. … so we have to give a sleep study again And then once they sleep, it helps with a lot of other things, it really does, with their depression, with their energy level, with having them be compliant with their appointments and medications, and pain…”  “We have this one patient. He’s having a real hard time managing his diabetes condition. When I go over to his house, he’s had blood glucose readings that just say “high” and when we take his blood glucose in the hospital, it’s 800, and normally he would kind of refuse transport, stuff like that. ...He wanted to remain far away from the hospital but meanwhile he’s out there and not managing his diabetes well. He got frostbite because he has diabetic neuropathy in his feet, couldn’t feel his feet. His feet started to have ulcers on them from this frostbite. It was…really bad, like second degree frost bite. Huge blisters, you know, just skin was sloughing off, and he didn’t even know until I came and assessed him, and then, from there, working with him we actually got him in-house right now. He’s in the facility and I think that wouldn’t have been accomplished from…other non-PIM team resources.”  “…Your average normal patient that comes in with diabetes you’re going to want them to have a hemoglobin A1C goal. These patients, that’s not feasible. Sometimes these patients, their biggest thing is housing, or their biggest thing is their fish, getting their fish tank taken care of before they’ll even take their insulin. A regular primary care role, they don’t have the staff or the time to be able to take a look at these patients to see how many times they’ve been in emergency room, what services they need, what worked in the past, what doesn’t work, to use harm reduction, to strategize, to see what didn’t work and what can work.” [also addresses SDOH, (intensive?) care management, what PIM does that PACT can’t]  “One of our patients, he has diabetes, so his vision has been affected by that…I don’t think he can really see how much Lantus he was drawing up in the syringe. ...We worked with his provider and we actually got him one of the easily injectable pens and a magnifying glass to make sure that he was taking the correct dosage of his long-acting insulin.”  “[A patient] had initially been talking about wanting to get his diabetes under control and all of the right things. But I said what really matters to you? And he said, well, I want to be able to see my granddaughter graduate in two years. I said then…that’s our vantage point that we’re going to come at this from the we want to be able to get you to that graduation ceremony, so how can we best support your health. ….And he said, you know, I really like that. It was just a shift…and he felt excited about it. ….I also really love the team approach. This is different from my experience with mental health because we had team meetings but it was never like this. This is a true team. We brainstorm. We talk about our own experiences and what we’re each seeing with the veteran and then we’re coming up with a plan based on what the veteran’s wanting and each of our areas of expertise.” |
| Patient or caregiver education | “…basically just seeing that patient go from not using the resources that were already there to being educated on the resources and not to lean on us, the PIM team, as their case manager but for the patient themselves to recognize that they have the power and the ability to manage their own care. And when I say, “manage their own care,” I’m not saying be their own doctors, but know how to use the system, how to talk to their doctor, how to talk to the RN case manager in primary care, where to go for all their needs. And one of the great things I think that we do is upon that initial home visit, we give them a folder. It’s called the PIM folder, PACT Intensive Management folder, and it’s full of all kind of information, a map of the VA, where to go, phone numbers, what to do with your old medications, how to reorder medications. …everything as a Veteran you kind of want to know.”  “…oxygen safety because a lot of patients are not—you know, they're smoking with the oxygen tank next to them and so it’s one of my concerns all the time, is to make sure that they're aware and taught, you know the correct safety procedures for O2…”  “...She is very overwhelmed with trying to care for her husband. She doesn’t quite understand what’s going on. Sometimes he may get upset with her because he’s overwhelmed too, and it presents a barrier in their relationship. So that’s an opportunity to…[build] that relationship with the caregiver... and talk about maybe some things that she could do to engage in self-care…because like the Veteran…has insulin and he has to take it, but he’s unable to give it to himself due to some of his other illnesses. So just educating the wife, the caregiver, on how to give the insulin...She called the nurse and the nurse was able to walk her through it over the phone. And that was very helpful because she began to trust us, and …then she felt more confident. And then being able to link her to the caregiver support program…being able to talk to other caregivers, she was able to see that she’s not alone, and that it’s not just her that’s going through it. …And it also helped their relationship…they’re not arguing as often. And when they come to the clinic they seem to be a little bit more at peace.” (SW) |
| Reliable Access to VA Staff & Services | "They can actually reach us is the biggest thing. Most people say every time they call the VA, they don’t get a call back or, “it’s hard to reach my doctor; it’s hard to get feedback.” I left a message for my social worker or my physician and I’ve not heard back in three days.” We’re easier to reach. We actually call back."  “I really think that the greatest success story is just the human touch and that now they’re connected [to providers]. These patients feel so happy that they have somebody that they can call that’s going to answer the phone, that’s going to call them back, that’s going to look at their blood pressure reading and say this is good, this is not, this is what you do. I mean at a VA this large and this many patients you just can’t get that. I mean, I was a social worker for 10 years in Primary Care…I probably had 12,000 patients combined, it would be two weeks before I could call a patient, even a patient I knew very well, back. You just can’t get that kind of same-day access outside of PIM”  “[Many high need patients] don’t feel that the VA is going to help them; they feel that we don’t care. They feel like we’re just sitting around and doing nothing. So just showing them that, no, we’re working, we’re following up. You can reach us whenever you need us has been very important in getting them engaged in their healthcare and showing them that we care just as much; we’re passionate about them getting better has been very helpful.”  “Oh, the biggest successes are patients, no matter how small their goals are, that their confidence has improved. I mean, I’ve had a lot of patients come in here very dejected, very pessimistic about their health overall. There’s no doubt that they have a lot of medical challenges, that they have a lot of medical issues going on concurrently but having their confidence and their hope raised and really empowering them by setting goals with them, by getting them to find some goals that resonate with them has really been eye-opening. I mean, they really have woken up to what can be done. *And the other thing is* *access to care has improved*, they’re able to get in with us when they need. So they really can improve that therapeutic relationship. I think that’s been the biggest positive with this program so far for us at least. To see people succeed that you thought they may not succeed. You don’t know who’s going to—that’s the thing, people will surprise you, people will surprise you with what they can do.”  [The first part of the above quote addresses patient need for a changed outlook including goals and motivation to work toward them; Note also the connections made by speaker among access, relationship-building, meaningful patient goals, and improvement in patient attitude (e.g., motivation, confidence, positive outlook)] |
| Strong patient or caregiver-healthcare professional and team member relationships; trust | “Just the other week I had to do one of the initial screening modules… on a patient that was inpatient. … he was grumpy because he had just lost his legs, and he’d become an amputee, and he was there rehabilitating and trying to go through physical therapy to get his prosthesis and whatnot. So, he was just not a happy man in general. But sharing with him [PIM program manager was also a Veteran] and asking him questions about his time in the service, and identifying similarities with mine, I think made him open up and have instant rapport…so it was cool to share experiences that were similar, and we had fun and laughed the whole time. And I learned so many things about him. And he was a patient that was somewhat reluctant to participate in PIM, but as I explained the people that would be giving him his direct care…understood and things like that, he was more inclined to participate…”  “…every time I meet with a patient I want to know a family member…that I can engage, just because inevitably the patient’s not going to respond, there might be concerns. It’s just always good to build a better support team with the patient…in my perspective build a better support team or connection with the patient’s informal supports. So, I ask the patient if it’s okay. I give the family member, caregiver, whomever my VA cell phone. I asked them to program it into the phone because often they’re the people who end up reaching out on the patient’s behalf. So, I found that really, really useful. I have a lot of wives programmed into my phones, like wives of veterans. So much so that, listen how crazy this is…we had a wife of a veteran who we’ve been working closely with. Then one of our patients who we really need to get out of his current apartment because he lacks capacity, but he wouldn’t let us… he wasn’t letting us contact the family. Finally, he’s like, “Call my one sister.” The sister’s like, “Call my other sister.” Three sisters down the line turned out to be the wife of our other veteran, who we’ve already been working with. …And we were like, “We love her.” She’s an amazing caregiver to—it’s actually her ex-husband… and she has been like getting things done with this veteran. So, it’s really been helpful in building the relationship with her… this is all working so much faster for the other veteran now.” [Also addresses communication issues]  “…we have one gentleman, younger Veteran. He’s about 29…and he expressed some concerns with the VA as a whole…just not trusting the VA…not being treated correctly, no one actually sitting down and listening to him, which is one of his biggest things. So, I took the opportunity to do just that, listen to him. …And I just sat there and listened to him, provided different ways that he could articulate himself when he’s meeting with the provider, trying to activate him so he could be more confident in talking to our doctor or talking to our nurse. And listening to some of the things that he was going through at home…”  Q: And was there any follow-up? Did you have a sense of what kind of effect that meeting had?”  A: “Well, yeah…after meeting with us and sitting down and seeing that we were going to do things differently, we were going to actually listen to him, he trusted our recommendations as far as like the pain medicines go. He’s in a lot of pain, but the PIM didn’t think that he needed any opiates and he trusted our recommendation. And so, what he did was he decided to do hypnosis for pain and I believe CBT for pain. And I believe that that rapport building was very important so that he was able to trust us.” [Could also be included in substance use]  “[One patient has been] one of those that’s a difficult one because he’s got a lot of anxiety, had a lot of substance abuse history, just to get him involved in his own care. And he’s also a fairly dependent personality so trying to move him towards the place of making his own appointments, cancelling his own appointments when he can't go, and doing those sorts of things. But we started at a place where he was kind of like resistant in some ways but built trust with him by helping him when he needed it and how he needed it. And it like, again, got him into a stable living environment. He was in a very unstable living environment.” [mental health issues; substance use; building trust/relationships] |
| Lifestyle change | “…what we anticipated was if we set everything up, had the right staff, had them appropriately trained, giving them the right time, we thought we would be able to melt butter. But, instead, thinking about human nature, it’s hard to change somebody’s behavior and habits and it’s hard to change people’s eating habits. it’s hard to give up smoking. It’s hard to exercise…Not to say we haven’t been successful; we’ve been very successful actually. But the amount of effort that goes into getting to that point is a lot more intensive and lot of work is required. It’s not as easy as anticipated…So it’s not like when you’re thinking, okay, I’ve got a patient and I’m just going to have a conversation with him and he’s going to quit smoking. No, it requires a lot of effort to get to that point where the person shares their story, where they talk about what really matters to them, and they share and then make the connection of why that is important to them and really have that conviction and then getting to that point where they see the connection and then they are willing to take a step to move in that right direction.” [could also fit in “relationship building” and “changed outlook”] |
| Changed outlook  (High-risk patients are often described as struggling with hopelessness, lack of confidence that anything can be done to improve their health, and these are connected with lack of motivation to do the work needed to improve their health, quality of life) | “I had a gentleman come in and his wife said, “look, Dr. [Name omitted], it sounds wonderful this PACT intensive management but I’m not sure, you know. He’s been through everything and he’s just basically given up hope…And the first two visits…I showed them the Wheel of Health, we talked about issues and value of his life and why does he want his health, what brings him a sense of joy, what brings him a sense of happiness and we went through all of that. And he really couldn’t articulate what that was. Every visit I met with him…and tried to really get to know him as a person in addition to addressing his medical needs. By the second or third visit he says to me, “Doc, I’ve decided, I want to do something with my life…as you know Doc, I like going fishing, I like being in the great outdoors. I can’t do that because my knees, I have osteoarthritis, it’s very painful, they told me I need knee replacement but I’m smoking and [I’m on oxygen] …and I can’t get that done.” And so, he says to me…, “I’m going to quit smoking. And in one year I’m going to be with my grandson, I’m going to be fishing with him, I’m going to show him how to hunt…This is what I want to do.” …I offered him smoking cessation program, he said no. I said, “What about the medications?” He said, “No, I’m going to do it myself.” .…He came back two months later, and…sure enough he had quit smoking. He was off the oxygen…he was cleared for surgery. …So now he’s really engaged in what he wants to do…. And the first day I asked him to quit smoking he said, “Doc, I lost everything in my life and the only thing I like is smoking; that’s all I’ve got left; I’ve lost everything.” And this is the same man six months later who would be talking a different tune. So that’s one example of an actual patient vignette that happened here.” |
| Difficulties in the home; improvements needed in the home environment | “We had another situation where the Veteran was—he’s wheelchair bound, he has a scooter, and his joystick kept breaking. So, the VA was pretty much saying it’s his fault; it’s your fault, you don’t know how to drive a scooter because this joystick, it keeps breaking. So, going to his home I was able to actually see that the doors were very narrow. The hallway was—I mean, I never seen a hallway so narrow. So, every time he would leave the living room to go to his bedroom it was so narrow he was hitting the joystick. And so, after so much it was becoming loose and after a while it just broke. And you can see all the indentions on the wall. So being able to provide that description of what’s going on in the community has been very beneficial in advocating for the Veterans.”  …They [PCPs] need to know that the Veterans have, say, a very difficult wound and they're having trouble taking care of it and the Veteran and the caregiver of the Veteran may not be able to even articulate that they need more wound bandages. …So, we can explain to the primary care physician what's going on with that. So, if they’re adding a fourth antibiotic, and we check [by visiting the home], and the real issue is that that person’s home is so dirty that the bandages are re-infecting the person, that could have a huge impact on their healthcare. Because no vulnerable person needs four antibiotics; It could really have an impact on the GI tract. So, again, lots of what we do is bring to light things that were just causing puzzlement and lack of response on the part of the person’s basic healthcare.”  “…I got a patient an air conditioner from prosthetics for his MS. I connected two to HISA. I’d never heard of that program, but I was in an IDT meeting and someone brought up HISA and I said, “What is HISA?” Anyway, so I'm like, “I have two Vets that—” so I got [them] connected to HISA. So one has a—they installed a chair lift and, yeah, it’s really nice because it took him an hour and fifteen minutes to get up his stairs but he didn’t want to move because he loved his landlord, he’s like a son to him, he said, and he was so very nice. And so we got a chair lift for him and then we got a shower chair for him. We got him a recliner, you know, that helps him get up.”” (RN,73:26-27)  “I was surprised that a Vet was actually crying when we got him connected to the stair glide—when I got him, you know—they went out to measure for the stair glide and to build him a ramp for the outside and the stair glide for the inside, and do all the changes. He called me crying, so that was sweet.”  “So we got there when he moved in and I mean literally helped him move some things around because he had nobody—and he’s a high fall risk—so just helped him even get set up in his apartment a little bit. I know that sounds very odd and nontraditional. So he’s been very much engaged” |
| Challenges navigating VHA care/services | “One thing I’ve noticed—and I’m a Veteran too so…I also experienced it, but you have all these changes that go on and you know nothing about it. And no one tells you nothing…You’re so confused. And…a lot of the Veterans [in PIM] have expressed that same frustration.”  “…it’s a big organization, the VA; very complex, very tough to navigate even if you have a sane, straight, healthy mind. So, you can imagine for some of our Veterans who have PTSD, depression, anxiety, some type of psychotic disorder, it’s very difficult just to navigate the world let alone the VA system which can be admittedly sometimes very convoluted. …I can talk one-on-one with the patients and really find out what's going on. …Maybe it’s a misunderstanding. Maybe it’s anxiety, PTSD…the hallmarks of are avoidance. So, you see someone not going to appointments, it may be because they're too anxious to walk into the building.”  “Some [patients] have said that they have been waiting for certain things for three and four months and nothing ever happened. They kind of gave up, and once we’ve gotten involved, they’ve actually seen results.” |
| Communication problems/Care Coordination | Like there was a man that we…He’s like 80 years old…He was walking around for weeks refusing to take a shower, in a hospital gown, and it was really sort of a mess. And…there was a big lack of communication because the care home workers were all Filipino, and he…didn't speak any Filipino. [And] they didn't understand what was the matter with him and what was driving him to be so irritable and difficult. So, we went into the [board and care] home and were able to work with the caregivers to better understand him, and for him to understand what their needs were, and to solve some of the communication problems. …And a lot of it was just…brute persistence… [the PIM] nurse…just went back over and over again till she got him to accept a shower from them. And…work out a medication system…sort of a compromise: She kept the key to the medication box, but they would bring the box in. …And, that was one of those where you walk in and you just see it, the problem that you also really have to work very specifically in that setting to keep that person functioning in that setting, which they were eventually able to do.”  “This actual particular Veteran had a certain type of brace in mind and he felt like he was trying to tell the doctor what he needed, and they didn’t understand him. Once we got involved [connecting with prosthetics], he got the ones he needed.”  “If they [a Veteran] missed their appointment or they missed a consult just calling them, following up. “Hey, I saw you missed your appointment with the podiatrist. Is everything okay? Can I help with coordinating this, can I reschedule?” …our nurse, she’s had to contact the specialty clinics quite often to reschedule appointments. So, just checking in with the Veterans and making sure that they’re okay before a crisis happens has been very helpful in getting them engaged and building that trust because that’s what we notice is the biggest thing is that a lot of the Veterans don’t trust the system… They don’t feel that the VA is going to help them; they feel that we don’t care.”  “We have found…patients who were supposed to follow-up with X, Y, Z clinic, they missed it for some reason and now they’ve been lost to follow-up. We found a multitude of things where patients are falling through the cracks and we can actually get them, because we’re all looking at the chart we are able to identify those and provide a higher quality of service.” |

Notes: Compiled by [redacted] Department of Veterans Affairs, Los Angeles: May 2019; CBT cognitive behavioral therapy, CPAP continuous positive airway pressure, HISA home improvements and structural alterations, IDT interdisciplinary team, PACT Patient Aligned Care Team, PCP primary care provider, PIM PACT Intensive Management, PTSD post-traumatic stress disorder, RN registered nurse, SW social worker, VA department of veterans affairs and veterans health administration

**Literature Review Search Strategies**

Database: PubMed

Date: 2/7/2020

Search Terms

(“high need patients” OR "frequent user" OR "frequent users" OR "frequent utilisation" OR "frequent utilization" OR "frequent utilizers" OR "heavy utilization" OR "heavy utilizer" OR "heavy utilizers" OR "high attender” OR "high attenders” OR "hyperuser" OR "hyperusers" OR "hyper-user" OR "hyper-users" OR "hyperutilization" OR "medically complex" OR "overutilization" OR "overutilisation" OR "recividism" OR "repeat user” OR "repeat users” OR "revolving door patient" OR "revolving door patients" OR "super utilizer" OR "super utilizers" OR "super utilizing” OR “Frequent flyer” OR “Frequent flyers” OR “high cost” OR “High costs” OR “high need” OR “high needs” OR “High risk” OR “High risks” OR “high utilizer” OR “High utilizers” OR “high utiliser” OR “High utilisers” OR “high utilizing" OR “high utilising" OR ”Complex” OR ”Frequent attender” OR ”Frequent attenders” OR ”Multi-morbid*” OR ”Multimorbid*” OR "Multiple Chronic Conditions"[Mesh] OR "Comorbidity"[Mesh] OR "multiple Chronic Condition" OR "multiple Chronic Conditions" OR "multiple medical Condition" OR "multiple medical Conditions" OR "multiple Chronic illness" OR "multiple Chronic illnesses" OR "multiple medical illness" OR "multiple medical illnesses")

AND

(Primary Health Care[Mesh] OR Physicians, Primary Care[Mesh] OR "primary healthcare" OR "primary health care" OR "Primary care" OR "PCP")

AND

(Meta-Analysis[Publication Type] OR systematic review[Filter] OR "systematic review" OR "systematic reviews" OR meta-analysis OR metaanalysis OR metanalysis OR meta-analyses OR metaanalyses OR metanalyses OR PRISMA)

OR

((need*[Title]) AND (multi-morbid* OR co-morbid*))

(need*[Title]) AND utilizer*

(need*[Title]) AND high-need patient*

(need*[Title]) AND complex patient*

((need*[Title]) AND patient*[Title]) AND high-risk[Title]

((need*[Title]) AND patient*[Title]) AND complex[Title]

((need*[Title]) AND patient*[Title/Abstract]) AND high-cost[Title]

**Literature Review Eligibility Criteria**

- Study types and data format
  - Systematic reviews, scoping reviews, evidence maps, or realist reviews of the literature addressing complex patients, high needs patients, high-cost, high utilizers (services or cost), super users, high-risk patients, and multi-morbid patients. Other reviews or opinion papers as well as reviews on other patient groups were excluded.
  - Empirical studies assessing the needs to complex patients, the needs of healthcare professionals caring for complex patients, or the needs of healthcare delivery organizations caring for complex patients. We used the author’s definition of *needs* and acknowledge that some needs may be more appropriately termed *wants* or *concerns*. Studies exclusively addressing other patient characteristics were excluded.
  - Empirical studies providing construct validity for the term ‘complex’ or ‘high need’ and studies defining the complexity of complex patients. Opinion papers not reporting empirical analyses were excluded.
  - Publications providing a taxonomy of interventions that have been used to address complex patients, high needs patients, high-cost, high utilizers (services or cost), super users, high-risk patients, and multi-morbid patients. Publications only reporting on a single intervention were excluded.
  - Publicly available tools or tool collections (e.g., reviews of measures) for complex patients, healthcare professionals, and organizations. Tools primarily aimed at other targets or content areas (e.g., not health care) were excluded
- Participants
  - Complex patients, high needs patients, high-cost patients, high utilizers (services or cost), super users, high-risk patients, multi-morbid patients.
- Setting
  - Material had to be relevant to primary care and information exclusively relevant to other care settings were excluded.

**Appendix Figure 1: Literature Review Flow Diagram**

Additional records identified through other sources
(n = 30)

Records identified through database searching
(n = 2,553)

Citations screened
(n = 2,583)

Excluded Citations (not on topic population, not a systematic review, not addressing needs) (n = 2,387)

Full-text articles excluded, with reasons

Exclude-Participants: n = 32

Exclude-Study Design: n = 32

Exclude-Setting: n = 11

Duplicate: n = 2

Full-text publications assessed for eligibility
(n = 196)

Background

(n = 32)

Included Studies

(78 studies reported in 87 publications)

## **Appendix Table 3: Literature Review Evidence Table Research Syntheses**

| **Publication**  **Type of review**  **Search date** | **Review topic**  **Issues not addressed in policy or research** | **Definition of high-need**  **Identified predictors of high need** | **Interventions included in the review**  **Unintended consequences**  **Highlighted issues** | **Patient needs**  **Healthcare professional needs**  **Tools** |
| --- | --- | --- | --- | --- |
| Baker, 2018^1^  Systematic review  May 2015 | Topic: Necessary components and appropriate intensity of effective care management interventions targeting multimorbidity  Issues: Need greater understanding of mental health in care management; need better methods for identifying patients who would benefit most from care management; need to dermine which components of interventions and the intensity is needed for which patient group | Definition: Adults with 2 or more chronic medical conditions; adults with at least 1 chronic medical condition & concurrent depression; adults identified based solely on high past or predicted healthcare utilization  Predictors: N/A | Interventions: Patient-focused, comprehensive care management intervention (areas of focus included some combination of self-management, healthcare system navigation, self-efficacy, symptom monitoring, symptom management, etc.) targeting the "whole" patient  Unintended consequences: N/A  Highlighted issues: Need greater understanding of mental health in care management; need better methods for identifying patients who would benefit most from care management; need to dermine which components of interventions and the intensity is needed for which patient group | Patient needs: N/A  Professional needs: N/A  Tools: N/A |
| Bleich, 2015^2^  Systematic review,Taxonomy  June 2014 | Topic: Care models for high need and high risk people with multiple chronic conditions or disabilities  Issues: Care and case management models are a more effective approach for reducing health care use & costs amoung high need high cost patients with multiple chronic conditions | Definition: Multiple chronic conditions (behavioral or mental health) or disabilities  Predictors: N/A | Interventions: Model type (care/case management, chronic disease self-management, disease management, nursing home, transitional care) and outcomes  Unintended consequences: N/A  Highlighted issues: Care and case management models more effective approach for reducing health care use & costs amoung HNHC patients with multiple chronic conditions | Patient needs: N/A  Professional needs: N/A  Tools: N/A |
| Boehmer, 2018^3^  Systematic review  July 2016 | Topic: Chronic care model, Minimally Disruptive Medicine in multimorbidity  Issues: Future publications should measure patient-centered outcomes such as treatment burden, quality of life, and functional status | Definition: Multimorbidity--the coexistence and interaction of multiple chronic conditions  Predictors: N/A | Interventions: Chronic care model and Minimally Disruptive Medicine approaches  Unintended consequences: N/A  Highlighted issues: Future publications should measure patient-centered outcomes such as treatment burden, quality of life, and functional status | Patient needs: N/A  Professional needs: N/A  Tools: N/A |
| Boult, 2009^4^  Systematic review : Scoping review,Taxonomy  May 2008 | Topic: Models of care for older persons with several chronic conditions  Issues: Medicare limits support for many of these programs-- many essential providers (non-physicians) are not eligible for payment through Medicare; most care that is not face-to-face cannot be reimbursed | Definition: Older persons with chronic conditions  Predictors: N/A | Interventions: Models: interdisciplinary primary care, care/case management, disease management, preventive home visits, outpatient geriatric assessment, pharmaceutical care, chronic disease self-management, proactive rehabilitation, caregiver support, transitional care, hospital-at-home, nursing home, prevention/management of delirium, comprehensive hospital care  Unintended consequences: N/A  Highlighted issues: Medicare limits support for many of these programs-- many essential providers (non-physicians) are not eligible for payment through Medicare; most care that is not face-to-face cannot be reimbursed | Patient needs: N/A  Professional needs: N/A  Tools: N/A |
| Bunn, 2018^5^  Systematic review : Scoping review  N/A (before 2018) | Topic: Successful components of interventions to facilitate shared decision making in patients with multiple health and social care needs  Issues: The need to define and evaluate the contribution of health care team members to the shared decision making approach; Family-centered approaches to shared decision making | Definition: Older people with complex health and care needs, for example, people with frailty, multi-morbidity and long-term conditions  Predictors: N/A | Interventions: Shared decision making or person-centered care/personalized care planning  Unintended consequences: N/A  Highlighted issues: The need to define and evaluate the contribution of health care team members to the shared decision making approach; Family-centered approaches to shared decision making | Patient needs: N/A  Professional needs: N/A  Tools: N/A |
| Butterworth, 2019^6^  Systematic review  August 2018 | Topic: Decision-making interventions in multi-morbidity  Issues: Researchers need to attempt to include vulnerable populations that are often excluded from studies such as those in end-of-life care, cognitive decline, long-term care facilities, and patients with language barriers | Definition: Multi-morbidity: 2+ occurring chronic conditions  Predictors: N/A | Interventions: Involving patients in decision-making, including- patient workshop, individual coaching, holistic patient review, multi-disciplinary practitioner training, and organization change  Unintended consequences: Involving patients in decision-making appear to make little to no difference in health-related quality of life  Highlighted issues: Researchers need to attempt to include vulnerable populations that are often excluded from studies such as those in end-of-life care, cognitive decline, long-term care facilities, and patients with language barriers | Patient needs: N/A  Professional needs: N/A  Tools: N/A |
| Coller, 2014^7^  Systematic review,Taxonomy  May 2013 | Topic: Potentially preventable hospitalizations and interventions to reduce hospitalizations  Issues: N/A | Definition: Children with medical complexity  Predictors: N/A | Interventions: Home visits, care coordination, chronic care management, continuity across settings (transitions of care)  Unintended consequences: N/A  Highlighted issues: N/A | Patient needs: N/A  Professional needs: N/A  Tools: N/A |
| Coventry, 2015^8^  Systematic review : Qualitative meta-synthesis  April 2015 | Topic: Experiences and self-management in patients with mental and physical multimorbidity  Issues: N/A | Definition: Patients with the co-existence of two or more chronic conditions, where one is not necessarily more central than the others  Predictors: N/A | Interventions: N/A  Unintended consequences: N/A  Highlighted issues: N/A | Patient needs: Dealing with bodily and emotional impact, chronic fatigue, decisions about medicine, minimizing side effects of medicines, marshaling relations between them and significant others, preservation of function, coping strategies, retaining independence, preserving self-identity  Professional needs: N/A  Tools: N/A |
| De Groot, 2003^9^  Systematic review : Critical review,Tool  September 2000 | Topic: Measures of co-morbidity and multi-morbidity  Issues: N/A | Definition: There is no consensus on the definition of [co-morbidity] terms.  Predictors: N/A | Interventions: N/A  Unintended consequences: N/A  Highlighted issues: N/A | Patient needs: N/A  Professional needs: N/A  Tools: Co-morbidity measures: Charlson Index, Cumulative Illness Rating Scale (CIRS), Index of Coexisting Disease (ICED), Kaplan Index are most reliable and valid.  Others include: Burden of Disease (BOD), Cornoni-Huntley index, disease count, Duke Severity of Il  https://www.sciencedirect.com/science/article/abs/pii/S0895435602005851 |
| Diederichs, 2011^10^  Systematic review,Tool  August 2009 | Topic: Instruments/indices to measure multi-morbidity  Issues: Heterogeneity of measures of multi-morbidity | Definition: Multi-morbidity: coexistence of 2+ chronic diseases  Predictors: N/A | Interventions: N/A  Unintended consequences: N/A  Highlighted issues: Heterogeneity of measures of multi-morbidity | Patient needs: N/A  Professional needs: N/A  Tools: Indices: Charlson Index, Comorbidity Symptoms Scale, Seattle Index of Comorbidity, Medication-based Disease Burden Index, KoMo Score, Index of Coexisting Diseases, Functional Comorbidity Index, Incalzi Index, Kaplan-Feinstein Index, Physiologic Index  https://academic.oup.com/biomedgerontology/article/66A/3/301/600233 |
| Edwards, 2017^11^  Systematic review,Taxonomy  March 2017 | Topic: Effectiveness of intensive primary care interventions targeting complex patients at high risk of hospitalization or death  Issues: An intuitive approach to caring for these vulnerable patients is to broaden the scope, increase the intensity, and improve the coordination of outpatient care, in the hope that this will lead to reduced hospital use and lower costs | Definition: Patients identified as high risk for hospital admission or death  Predictors: N/A | Interventions: Primary care replacement (home-based and clinic-based), primary care augmentation  Unintended consequences: Adding more providers to care can increase discontinuity for patients and increase care coordination burden  Highlighted issues: An intuitive approach to caring for these vulnerable patients is to broaden the scope, increase the intensity, and improve the coordination of outpatient care, in the hope that this will lead to reduced hospital use and lower costs | Patient needs: N/A  Professional needs: N/A  Tools: N/A |
| Fraccaro, 2015^12^  Systematic review  January 2014 | Topic: Clinical decision making for multimorbid patients  Issues: Lack of patient-centered approaches; lack of rigorous evaluations; patients with multi-morbidity often excluded from clinical trials | Definition: Multimorbidity: “any combination of chronic disease with at least 1 other disease (acute or chronic) or bio-psychosocial factor (associated or not) or somatic risk factor."  Predictors: N/A | Interventions: N/A  Unintended consequences: Merging guidelines and automation of care processes may lead to harmful clinical actions  Highlighted issues: Lack of patient-centered approaches; lack of rigorous evaluations; patients with multi-morbidity often excluded from clinical trials | Patient needs: N/A  Professional needs: N/A  Tools: N/A |
| Gobeil-Lavoie, 2019^13^  Systematic review : Systematic thematic analysis  October 2018 | Topic: Characteristics of self-management among patients with chronic diseases and complex healthcare needs  Issues: Healthcare providers can use results to better adapt their interventions to meet needs of these patients | Definition: Adults with complex healthcare needs (multimorbidity, vulnerability, complexity and frequent use of health services)  Predictors: N/A | Interventions: Self-management  Unintended consequences: N/A  Highlighted issues: Healthcare providers can use results to better adapt their interventions to meet needs of these patients | Patient needs: Need for prioritization of self-care; need to address lack of motivation and higher risk of depression; need to address increased risk of presenting poor self-efficacy; need to address increased risk of receiving conflicting information on management of diseases by numerous health rofessionals; need to identify opportunities to use knowledge and personal experiences acquired in past and apply to better manager health  Professional needs: N/A  Tools: N/A |
| Haroun, 2016^14^  Systematic review  August 2015 | Topic: Interventions for frequent attenders of GPs  Issues: Frequent attending does not always persist, so applying a follow-up period of at least a year could be beneficial | Definition: We accepted all definitions of the term ‘frequent attender’; definitions varied widely-- using percentiles of attendance distribution, cut-off number of consultations, and one study using a "healthcare utilization algorithm"  Predictors: N/A | Interventions: Health education, enhanced care, group visit with GP nurse, offering referrals, depression management, problem solving treatment, disclosure of emotional events, multi-step intervention, GP education, acupuncture, mindfulness training, status consultation  Unintended consequences: N/A  Highlighted issues: Frequent attending does not always persist, so applying a follow-up period of at least a year could be beneficial | Patient needs: N/A  Professional needs: N/A  Tools: N/A |
| Hohmann, 2019^15^  Systematic review  December 2018 | Topic: Patient perspectives of care coordination for cancer and multiple chronic conditions  Issues: N/A | Definition: Multiple chronic conditions: one or more chronic comorbid condition of any kind; Patients with both cancer and multiple chronic conditions were the focus of this review  Predictors: N/A | Interventions: N/A  Unintended consequences: N/A  Highlighted issues: N/A | Patient needs: Need for improved communication between providers, improved physician knowledge of individual patient needs and preferences, defining provider care roles and expectations, access to care information, need for a designated contact-person if patients have questions about their care, individualized patient care  Professional needs: N/A  Tools: N/A |
| Hohmann, 2020^16^  Systematic review  December 2018 | Topic: Provider's views on care coordination for cancer and multiple chronic conditions  Issues: N/A | Definition: Patients with cancer AND multiple chronic conditions  Predictors: N/A | Interventions: N/A  Unintended consequences: N/A  Highlighted issues: N/A | Patient needs: N/A  Professional needs: More coordination between PCPs and oncologists, differing viewpoints on shared care, some oncologists believe PCPs cannot adequately provide cancer follow-up care, expedited referrals to specialists, specialist-provided patient letter, lack of information  Tools: N/A |
| Hudon, 2019^17^  Systematic review,Taxonomy  September 2017 | Topic: Case management intervention for frequent users with chronic disease  Issues: Clinicians and policy makers should focus on case-finding processes | Definition: Did not provide specific definition  Predictors: N/A | Interventions: Case management interventions  Unintended consequences: N/A  Highlighted issues: Clinicians and policy makers should focus on case-finding processes | Patient needs: N/A  Professional needs: N/A  Tools: N/A |
| Huntley, 2012^18^  Systematic review,Tool  December 2009 | Topic: Multi-morbidity measures  Issues: N/A | Definition: Multi-morbidity: the co-occurrence of multiple diseases or multiple medical conditions within one person  Predictors: N/A | Interventions: N/A  Unintended consequences: N/A  Highlighted issues: N/A | Patient needs: N/A  Professional needs: N/A  Tools: Disease counts, Chronic Disease Score (CDS/RxRisk), Adjusted Clinical Groups (ACG) System, Charlson Index, Cumulative Index Illness Rating Scale, Duke Severity of Illness Checklist (DUSOI)  http://www.annfammed.org/content/10/2/134.short |
| Johnston, 2019^19^  Systematic review : Systematic reviews of systematic reviews  February 2017 | Topic: Defining and measuring multi-morbidity  Issues: No common approach to measuring or defining multi-morbidity; researchers should be explicit about definitions and give a rationale for their choice | Definition: Multiple definitions in multiple papers. Often included a cut-off of two or more conditions. Definitions varied regarding duration of condition and severity of condition.  Predictors: N/A | Interventions: N/A  Unintended consequences: N/A  Highlighted issues: No common approach to measuring or defining multi-morbidity; researchers should be explicit about definitions and give a rationale for their choice | Patient needs: N/A  Professional needs: N/A  Tools: Measures included disease counts and weighted indices: Charlson Index, the Cumulative Illness Rating Scale (CIRS), the Index of Coexistent Disease (ICED), the Adjusted Clinical Groups (ACG) System and the Duke Severity of Illness |
| Kastner, 2018^20^  Systematic review,Taxonomy  December 2017 | Topic: Interventions for multi-morbidity  Issues: Clinical practice guidelines lack information on management of multi-morbidity | Definition: Multi-morbidity: 2+ high-burden chronic conditions  Predictors: N/A | Interventions: Care coordination, information and health technology, education to patients and/or providers, disease management, self-management, cognitive-behavioral  Unintended consequences: N/A  Highlighted issues: Clinical practice guidelines lack information on management of multi-morbidity | Patient needs: N/A  Professional needs: N/A  Tools: N/A |
| Latour, 2007^21^  Systematic review  June 2005 | Topic: Post-discharge nurse-led care management  Issues: Need for studies with clearly defined measures of complexity or frailty.  Additional research on other health care systems.  Evaluation of the cost effectiveness of case management in complex patients. | Definition: Ambulatory patients over 18 years of age and defined as complex; patients with acute or chronic medical condition(s) and described other vulnerabilities, such as (psychiatric) comorbidity; frail elderly people; and patients with social problems, reduced f  Predictors: NA | Interventions: Nurse-led case management (assessment of needs, development of a comprehensive service plan, arrangement of service delivery, monitoring and assessment of services, evaluation, and follow-up)  Unintended consequences: Added expense of case manager  Highlighted issues: Need for studies with clearly defined measures of complexity or frailty.  Additional research on other health care systems.  Evaluation of the cost effectiveness of case management in complex patients. | Patient needs: N/A  Professional needs: N/A  Tools: N/A |
| Le Reste, 2013^22^  Systematic review  December 2010 | Topic: Multi- morbidity criteria and how it can be used to create definition  Issues: Having a more comprehensive definition can help to better focus research, especially for areas such as quality of care and cost of care | Definition: Themes were chronic disease, acute disease, biopsychosocial factors and somatic risk factors, coping, burden of disease, healthcare consumption, disability, quality of life, frailty, social network, health outcomes  Predictors: - Any combination of chronic disease with at least one other disease (acute or chronic) or bio- psychosocial factor (associated or not) or somatic risk factor.  - Any biopsychosocial factor, any somatic risk factor, the social network, the burden of diseas | Interventions: N/A  Unintended consequences: N/A  Highlighted issues: Having a more comprehensive definition can help to better focus research, especially for areas such as quality of care and cost of care | Patient needs: N/A  Professional needs: N/A  Tools: N/A |
| Liddy, 2014^23^  Systematic review : Systematic qualitative thematic synthesis  September 2013 | Topic: Perspectives on self-management of patient with multiple chronic conditions perspectives  Issues: Medical task management and patient education is often the focus of the medical community, but patients endorsed problems not with that but with difficulties in dealing with physical and emotional symptoms, and confusing information provided by multiple h | Definition: Multi-morbidity: 2+ simultaneous diseases or medical conditions in an individual  Predictors: N/A | Interventions: N/A  Unintended consequences: N/A  Highlighted issues: Medical task management and patient education is often the focus of the medical community, but patients endorsed problems not with that but with difficulties in dealing with physical and emotional symptoms, and confusing information provided by multiple h | Patient needs: Managing pain, physical, and emotional symptoms, managing depression, lack of motivations or self-discipline, lack of energy, fear for personal health and of death, changing cognitive approach to illness, relinquishing control, prioritizing conditions, lack of social support, lack of financial resources, therapeutic alliance (contradictory knowledge, poor access), challenges with medication, finding common ground between patient and provider, tailored written information and care plans, assistance with locating resources, better access to mental health resources  Professional needs: N/A  Tools: N/A |
| Mangin, 2016^24^  Systematic review,Tool  January 2015 | Topic: Tools for patient treatment preferences  Issues: N/A | Definition: No definition provided  Predictors: N/A | Interventions: N/A  Unintended consequences: N/A  Highlighted issues: N/A | Patient needs: N/A  Professional needs: N/A  Tools: Patient Goal Priority Questionnaire (PGPQ) and Patient Goal Priority List (PGPL), Pre-CS Survey, Control Preference Scale (CPS), Adaptive Conjoint Analysis (ACA), Likert Scale, Priority  http://dx.doi.org/10.1136/bmjopen-2015-010903 |
| Marcoux, 2017^25^  Systematic review : Scoping review,Tool  July 2016 | Topic: Identification of short, valid screening tools to identify adult patients with complex health needs  Issues: Need to evaluate screening tools for identifying patients with complex needs at risk of becoming high users of healthcare services and for whom a CM intervention could be beneficial | Definition: Adult patients of all ages  Predictors: Prior healthcare utilization, medical conditions (self-reported) medical diagnosis, medications, emotional status/mental health, socioeconomic condition | Interventions: N/A  Unintended consequences: N/A  Highlighted issues: Need to evaluate screening tools for identifying patients with complex needs at risk of becoming high users of healthcare services and for whom a CM intervention could be beneficial | Patient needs: N/A  Professional needs: N/A  Tools: Collection of screening tools; INTERMED self-assessment can target adults of all ages, completed in less than 15 minutes  https://doi.org/10.1371/journal.pone.0188663 |
| Marengoni, 2011^26^  Systematic review  November 2010 | Topic: Definitions and associations of multi-morbidity  Issues: Research is needed on genetic, lifestyle, and contextual factors that contribute to multi-morbidity | Definition: Multi-morbidity: three major definitions-- number (usually 2+ concurrent diseases in an individual), cumulative indices which evaluate number and severity of diseases, simultaneous presence of diseases and cognitive/physical limitations  Predictors: Older age, female gender, low socioeconomic status, elderly living home, lower education, food insufficiency, living alone | Interventions: N/A  Unintended consequences: N/A  Highlighted issues: Research is needed on genetic, lifestyle, and contextual factors that contribute to multi-morbidity | Patient needs: N/A  Professional needs: N/A  Tools: N/A |
| Moe, 2017^27^  Systematic review  October 2014 | Topic: Interventions to decrease emergency department visits by adult frequent users  Issues: Inconsistency in frequent use definitions and methodologies | Definition: Frequent users of emergency departments  Predictors: N/A | Interventions: Case management, care plans, diversion strategies, printout case notes, social work visits  Unintended consequences: N/A  Highlighted issues: Inconsistency in frequent use definitions and methodologies | Patient needs: N/A  Professional needs: N/A  Tools: N/A |
| Ng, 2018^28^  Systematic review,Tool  January 2017 | Topic: Identifying multimorbidity with data analytic methods  Issues: Comparison of multimorbidity patterns can only be done is the same analytic methods are used for grouping conditions, so studies should report detailed methodology when describing patterns of multimorbidity | Definition: Multimorbidity: co-occurrence of 2 or more health conditions in a person  Predictors: N/A | Interventions: N/A  Unintended consequences: N/A  Highlighted issues: Comparison of multimorbidity patterns can only be done is the same analytic methods are used for grouping conditions, so studies should report detailed methodology when describing patterns of multimorbidity | Patient needs: N/A  Professional needs: N/A  Tools: Analytic methods for identifying multimorbid condition groups: factor-analysis, hierarchical-clustering, unified-clustering algorithm, multiple correspondence analysis, network and cluster analyses  https://academic.oup.com/ije/article/47/5/1687/5054718 |
| Pillay, 2014^29^  Systematic review,Tool  N/A (before 2014) | Topic: Measures of quality of care in multimorbidity  Issues: Many measures are disease- specific | Definition: Multi-morbidity: 2+ chronic conditions in an individual  Predictors: N/A | Interventions: N/A  Unintended consequences: N/A  Highlighted issues: Many measures are disease- specific | Patient needs: N/A  Professional needs: N/A  Tools: Measures: Structure (continuity of care), Process (Recognition of condition, initiation of care, treatment intensification, recommended care process), Outcome (Intermediate outcome, symptom measures, functional outcomes, appropriate healthcare used, patie  https://www.racgp.org.au/download/Documents/AFP/2014/March/201403pillay.pdf |
| Poitras, 2018^30^  Systematic review : Scoping review,Taxonomy  2016 | Topic: Effective elements of interventions  Issues: Future research should include the innovative but less frequently reported elements in interventions | Definition: Multimorbidity: presence of multiple chronic or long-term conditions, including physical and mental diseases  Predictors: N/A | Interventions: Intervention elements: 1.) Supporting decision processes and evidence based practice, 2.) patient centered approaches, 3.) supporting self-management, 4.) providing care management, 5.) enhancing interdisciplinary team approach, 6.) training for providers, 7.) interrogating information technology  Unintended consequences: N/A  Highlighted issues: Future research should include the innovative but less frequently reported elements in interventions | Patient needs: N/A  Professional needs: N/A  Tools: N/A |
| Rosbach, 2017^31^  Systematic review : Review of qualitative research  June 2016 | Topic: Patient experiences of burden of treatment  Issues: Issues in recruitment/sampling since patients who are most burdened by disease might not be able to participate in voluntary research | Definition: Multi-morbidity: 2+ chronic conditions coexisting  Predictors: N/A | Interventions: N/A  Unintended consequences: N/A  Highlighted issues: Issues in recruitment/sampling since patients who are most burdened by disease might not be able to participate in voluntary research | Patient needs: Burden of treatment components: time spent on travel, arranging appointments, contradictory advice, multiple appointments, time off work, paperwork, wait to obtain treatment, communication with professionals, coordinating medication, medication interfering with activities, side effects, stigmatized because of medication, changing or obtaining medication, using medical equipment, altering diet, exercising, quitting smoking, paying for medication and health insurance, learning about condition, navigating healthcare system, self-monitoring of health, being a burden to friends/family  Professional needs: N/A  Tools: N/A |
| Sasseville, 2018^32^  Systematic review : Scoping review  July 2017 | Topic: Outcomes in multimorbidity interventions  Issues: Lack of patient-reported outcomes relevant for decision-making; smoking and alcohol consumption not addressed in any studies included in this SR | Definition: Multi-morbidity: 2+ chronic diseases  Predictors: N/A | Interventions: Chronic disease management intervention, including personalized care, goal assessment, self-management support, follow-ups, integrated care, monthly monitoring, transitional care, coordination of providers, support for caregivers, enhanced access to community services, collaborative care, management of drug treatment, education and counseling, phone follow-ups, newsletters, provider eduction, occupational therapy, structured visits, treatment targets, care plans, motivational approach, weekly community-based meetings, telehealth intervention, home assessment,  Unintended consequences: N/A  Highlighted issues: Lack of patient-reported outcomes relevant for decision-making; smoking and alcohol consumption not addressed in any studies included in this SR | Patient needs: N/A  Professional needs: N/A  Tools: N/A |
| Singh, 2016^33^  Systematic review : Scoping review  July 2014 | Topic: Extent and quality of literature regarding mobile health apps for high-need, high-cost populations  Issues: Limited early results support the use of mobile health apps for HNHC patients | Definition: Older adults; individuals with specific chronic conditions; physchologically or mentally vulnerable with specific diagnoses; individuals with physical handicaps or disabilities; socially vulnerable  Predictors: N/A | Interventions: Mobile health apps to treat high-need, high-cost populations  Unintended consequences: Not all apps found to be available to the public  Highlighted issues: Limited early results support the use of mobile health apps for HNHC patients | Patient needs: N/A  Professional needs: N/A  Tools: Various mobile health apps (not specified other than iOS or Android compatible) |
| Sinnott, 2015^34^  Sinnott, 2013^35^  Systematic review,Tool  September 2012 | Topic: GP perspectiveson the management of patients with multimorbidity, medication management  Issues: Limited evidence on which behavioral change techniques are effective in specific settings, multi-morbidity not a MeSH term and lack of consensus on definition | Definition: Multimorbidity: presence of 2 or more chronic (long-term) conditions  Predictors: N/A | Interventions: N/A  Unintended consequences: N/A  Highlighted issues: Limited evidence on which behavioral change techniques are effective in specific settings, multi-morbidity not a MeSH term and lack of consensus on definition | Patient needs: N/A  Professional needs: GPs have a feeling of professional isolation when dealing with multimorbid patients, disorganization and fragmentation of healthcare system, inadequacy of evidence-based guidelines, challenges in providing patient-centered rather than disease-focused care  Tools: MultimorbiditY COllaborative Medication Review And DEcision Making (MY COMRADE)  https://link.springer.com/article/10.1186/s13012-015-0322-1 |
| Smith, 2012^36^  Smith, 2017^37^; Smith, 2016^38^  Systematic review,Taxonomy  April 2011 | Topic: Effectiveness of interventions  Issues: N/A | Definition: Multi-morbidity: 2+ chronic conditions in an individual  Predictors: N/A | Interventions: Intervention elements: care manager, structured visits, telephone contact, patient care plans, enhanced multi-disciplinary team, home assessments, provider education, patient education, self management support, drug review, treatment targets, electronic registry to track patient progress, support for self care, pharmaceutical patient care plan, training of care coordinators, diet and exercise intervention, occupational therapy, physiotherapy, patient problem-solving techniques, patient "coaches"  Unintended consequences: Interventions more likely to be successful when targeting particular risk factors or functional difficulties  Highlighted issues: N/A | Patient needs: N/A  Professional needs: N/A  Tools: N/A |
| Smits, 2009^39^  Systematic review  November 2006 | Topic: Interventions  Issues: Using healthcare utilization may not adequately study FAs, as patients often change their health-seeking behaviors within 2-3 years | Definition: Frequent attenders: no specific definition but this SR provides definitions used by included publications  Predictors: N/A | Interventions: Depression management programme (DMP), Diagnostic Interview Schedule (DIS) by psychiatrist, brief educational programme, status consultation by GP, psychiatric interventions focusing on undiagnosed psychiatric morbidity  Unintended consequences: N/A  Highlighted issues: Using healthcare utilization may not adequately study FAs, as patients often change their health-seeking behaviors within 2-3 years | Patient needs: N/A  Professional needs: N/A  Tools: N/A |
| Soril, 2015^40^  Systematic review, Taxonomy  January 2015 | Topic: Interventions to reduce frequent visits to the emergency department  Issues: N/A | Definition: Frequent emergency department use  Predictors: N/A | Interventions: Case management, individualized care plans, information sharing  Unintended consequences: N/A  Highlighted issues: N/A | Patient needs: N/A  Professional needs: N/A  Tools: N/A |
| Soril, 2016^41^  Systematic review  September 2015 | Topic: Characteristics of frequent users of the emergency department  Issues: N/A | Definition: Frequent users of emergency departments  Predictors: Across healthcare systems, frequent emergency department users were more likely to be older, female, and have a mental health diagnosis, previous hospitalizations and high primary care use were associated with future frequent emergency department use in s | Interventions: N/A  Unintended consequences: N/A  Highlighted issues: N/A | Patient needs: N/A  Professional needs: N/A  Tools: N/A |
| Stokes, 2015^42^  Systematic review  April 2014 | Topic: Case management effectiveness  Issues: Definitions and measures of multi-morbidity should be more consistent; most evidence on case management is from high-income, Western settings and may not be applicable to other settings | Definition: Multi-morbidity: multiple long-term conditions  Predictors: N/A | Interventions: Case management, different models and components; community based multi-disciplinary teams, 24 hour phone availability, GP/case manager assessment and follow-up, disease-specific stream, education, self-management, integrating case management into daily life, care plans, coordinating services, counseling support, risk-stratification of patients, weekly team meetings to problem solve, telephone case management, fulfilling unmet social and medical needs, home-visits, coaching and monthly monitoring  Unintended consequences: N/A  Highlighted issues: Definitions and measures of multi-morbidity should be more consistent; most evidence on case management is from high-income, Western settings and may not be applicable to other settings | Patient needs: N/A  Professional needs: N/A  Tools: N/A |
| Vermunt, 2017^43^  Systematic review  November 2015 | Topic: Interventions with collaborative goal-setting  Issues: N/A | Definition: Frequent attenders older than 14 years old  Predictors: N/A | Interventions: PrefCheck (GP training session for priority setting consultations, computer aided geriatric assessment, independent problem importance rating), Guided Care (Nurse education program, customized EHR, patient assessment, chronic disease self-management, monitoring, coaching, transition coordination, educating and supporting caregivers, facilitating access to community resources), Helping Older People Experience Success (HOPES; community living skills, skills training, health management), Collaborative Care Model (Psychological therapy, stepped care, collaborative meetings), Integrated Systematic Care for Older People (GP and nurse training for integrative care, wishes and expectations of patient explored together).  Unintended consequences: N/A  Highlighted issues: N/A | Patient needs: N/A  Professional needs: N/A  Tools: N/A |
| Violan, 2014^44^  Systematic review  July 2013 | Topic: Determinants, patterns, and prevalence of multi-morbidity  Issues: Huge variation in prevalence (<15% to >95%), sample selection criteria, and methods for estimating multi-morbidity; lack of reliable methodological standards for identifying multi-morbidity; need more research on clustering of conditions | Definition: Multi-morbidity: Simultaneous presence of more than 1 health condition in an individual  Predictors: Age, gender, socioeconomic status, mental health disorders; grouped clusters of cardio-metabolic conditions, anxiety-depression, and pain | Interventions: N/A  Unintended consequences: N/A  Highlighted issues: Huge variation in prevalence (<15% to >95%), sample selection criteria, and methods for estimating multi-morbidity; lack of reliable methodological standards for identifying multi-morbidity; need more research on clustering of conditions | Patient needs: N/A  Professional needs: N/A  Tools: N/A |
| Welzel, 2017^45^  Systematic review  November 2016 | Topic: Definitions and associations of frequent attenders  Issues: Might be differences between short-term and long-term FAs; inconsistencies in defining FAs | Definition: Frequent attender- someone who attends primary care practices on a regular basis and exceeds a certain number of visits within a given time interval; this SR identified various definitions of FAs, most included studies used a proportional approach  Predictors: Number or severity of somatic diseases, mental illness/psychological distress, medical prescriptions, low social support, age, gender, living alone, lower quality of life, # of superficial contacts, frequent attendance out-of-hours | Interventions: N/A  Unintended consequences: N/A  Highlighted issues: Might be differences between short-term and long-term FAs; inconsistencies in defining FAs | Patient needs: N/A  Professional needs: N/A  Tools: N/A |
| Willadsen, 2016^46^  Systematic review  October 2013 | Topic: Defining multi-morbidity  Issues: Large variation in definition of multi-morbidity, including number and types of conditions | Definition: 2+ simultaneous chronic conditions in an individual ; multiple definitions in various articles  Predictors: Risk factors: hypertension, osteoporosis, hypercholesterolemia, obesity, overweight | Interventions: N/A  Unintended consequences: N/A  Highlighted issues: Large variation in definition of multi-morbidity, including number and types of conditions | Patient needs: N/A  Professional needs: N/A  Tools: Indices and databases: Charlson Comorbidity Index (CCI), Clinical Classification Software (CCS), Cumulative Illness Rating Scale (CIRS), Adjusted Clinical Groups (ACG), Aggregated diagnosis groups, medication-based Rx-MG, Expanded Diagnosis Clusters (EDC) |
| Yardley, 205^47^  Systematic review  August 2013 | Topic: Successes and failures in healthcare delivery and learning  Issues: Future research should identify meaningful markers or success that match with patient and clinician priorities | Definition: Multimorbidity: co-occurrence of two or more conditions, where one is not necessarily more central  Predictors: N/A | Interventions: Educational alliances, Beacon practices, communities of practice, ExBL, Breakdowns, Developmental space, Guided Care, Patient-Centered Medical Homes, CARE approach, Chronic Illness Care Plans, Chronic Care Model, Self-management support five A's, shared decision-making, transformative leaning, response shift, education-centered medical home  Unintended consequences: N/A  Highlighted issues: Future research should identify meaningful markers or success that match with patient and clinician priorities | Patient needs: Personalized concepts of success aren't always compatible with clinical markers, coping with multi-morbidity with proactive behavior, individual levels of ability to engage often fluctuate, time constraints, targets mismatched to patient needs or preferences  Professional needs: Changes in attitude required, supporting trainees to provide meaningful care, good interpersonal relationships and trust, providers frustrated with patients lack of readiness to change, success should be flexible, personalized and changeable  Tools: N/A |
| Yurkovich, 2015^48^  Systematic review,Tool  September 2012 | Topic: Comorbidity indices  Issues: N/A | Definition: Co-morbidity: total burden of illnesses unrelated to the principal diagnosis  Predictors: N/A | Interventions: N/A  Unintended consequences: N/A  Highlighted issues: N/A | Patient needs: N/A  Professional needs: N/A  Tools: Two categories of indices: 1.) Using ICD codes, 2.) using medication/pharmacy data. Indices include adaptations of the Charlson Index, the Elixhauser comorbidity index, Chronic disease score, RxRisk, and Medication-Based Disease Burden Index. Diagnosis-  https://www.sciencedirect.com/science/article/abs/pii/S0895435614003576 |
| Zullig, 2016^49^  Systematic review,Tool  January 2014 | Topic: Conceptual model of patient complexity  Issues: Need to understand drivers and causal pathways to design interventions | Definition: Patient complexity (e.g., multiple chronic conditions and contextual factors)  Predictors: N/A | Interventions: N/A  Unintended consequences: N/A  Highlighted issues: Need to understand drivers and causal pathways to design interventions | Patient needs: N/A  Professional needs: N/A  Tools: Cycle of complexity framwork  DOI: 10.1007/s11606-015-3512-2 |

Notes: DOI digital object identifier, GP general practitioner, N/A not available

## **Appendix Table 4: Literature Review Evidence Table Validity Studies**

| **Author, year** | **Sample characteristic** | **Definition or operationalization of high-need** | **Predictor variables** | **Other relevant data providing content, construct, or face validity** |
| --- | --- | --- | --- | --- |
| Belanger, 2019^50^ | Retrospective cohort study of all Medicare beneficiaries in 2014 who survived until the end of the year (n=54,717,039)  USA | High need: 2 or more complex conditions, 6 or more chronic conditions, acute or post-acute health services utilization, indicators or frailty, complete dependency in mobility or in any activities of daily living in post-acute care assessments, hospitalization, mortality, days in community, Medicare expenditures | Chronic conditions, healthcare utilization, functional impairment | High-need patients had mortality rates 7.1 times higher (16.23% vs 2.27%) and hospitalization rates 3.4 times higher (40.69 vs 12.03) compared to other beneficiaries. Competing high-need definitions all had good specificity (≥  0.88). Having 3 or more Hierarchical Chronic Conditions yielded a good positive predictive value for hospitalization, at 0.50, but only identified 19.71% of beneficiaries hospitalized and 28.46% of decedents that year as high need, as opposed to 33.92% and 51.98% for the new definition. |
| Buja, 2018^51^ | Patients 65+ characterized by complex health needs  Italy | Patients with complex healthcare needs: persons whose care requirements are numerous and costly Multimorbidity: co-occurrence of multiple chronic or acute diseases without one disease necessarily being primary | Five disease clusters identified in exploratory latent class analysis (BIC 40): metabolic-ischemic heart diseases, neurological and mental disorders, cardiac diseases, respiratory conditions, and neoplasms | N/A |
| Hayes, 2016^52^ | 2009-2011 Medical Expenditure Panel Survey population; non institutionalized adult civilian US population  USA | High needs: 3 or more chronic diseases and a functional limitation in ability to care for themselves or perform routine daily tasks | Having a functional limitation; older; female; white; less educated; low income; publicly insured; fair or poor self-reported health | High needs cohort had more emergency department visits, visits with doctor or home health care; higher spending and persistent high-cost |
| Haywood, 2018^53^ | 18+, and have had at least 2 hospital/ED encounters within 180 days  USA | High-need, high-cost: patients are frequently 65+, publicly insured with multiple chronic conditions and high rates of healthcare utilization | Oschsner high risk: patients having greater or equal to three 3 chronic diseases, AND greater than or equal to 2 hospital admissions, OR greater than or equal to 2 ED visit in the last 12 months, AND greater than or equal to 10 active medications AND one of the following diagnoses: depression, impaired cognition, or fall in the last 12 months. | High-risk patients had higher odds of 90-day hospital readmissions but not 90-day ED visits compared to non-high risk. |
| Matzer, 2012^54^ | Emergency department patients  Austria | INTERMED score > 20 | Biopsychosocial case complexity | Cluster analysis identified highly complex patient as a cluster that differed significantly from the other clusters as they had visited the emergency room more often within the last year and lived alone more frequently |
| Nuti, 2019^55^ | Patients insured by a Medicaid managed care organization admitted to hospital at least 3 times within 12-month period, OR admitted at least twice within same period and at least 1 serious mental health condition as primary diagnosis.  USA | High-need, high-cost: defined based on utilization of care | Based on machine learning algorithms, the two largest clusters were depression and other mood disorders, and 12/25 of top clusters were primarily mental health and substance use conditions. | The largest patient subgroups based on clinical characteristics were characterized by mental and behavioral conditions; an unexpected patient population was patients with pregnancy-related complications |
| Sarnak, 2016^56^  Osborn, 2014^57^ | Surveys across 9 countries  Multiple countries | High need: Age 65 and older with at least three chronic conditions or a functional limitation in activities of daily living, like dressing or bathing | N/A | Use of healthcare: across all countries, high-need patients use more health care compared with other older adults |
| Schickedanz, 2019^58^  Rogers, 2020^59^ | 34,225 patients (7,107 in intervention group & 27,118 in control group)  USA | 18 years or older and predicted to be in top 1% of health care utilization in KPSC | N/A | 53% screened positive for at least one social need |
| Smeets, 2020^60^ | 12,602 HNHC patients from 63 general practices in Netherlands  Netherlands | High-need, high-cost, chronically ill: chronically ill in top 10% of care utlizers and/or with multimorbidity plus above-average care utlization | Demographic (sex, age, household position); biomedical (type of chronic condition); socioeconomic (housing situation, source of income, # of peole with individual income in a household, household dependence); care utilization (pharmaceutical costs) | 4 classes identified in latent class analysis: 1 - older adults living with partner; 2 - older adults living alone; 3 - middle-aged employed adults with family; 4 - middle-aged adults with social welfare dependency |

## **Appendix Table 5: Literature Review Evidence Table Needs Assessments**

| **Study ID**  **Country** | **Sample**  **High need population and definition** | **Patient needs** | **Professional needs** |
| --- | --- | --- | --- |
| Bhattacharyya, 2019^61^  Canada | Interviews with 8 patient-caregiver pairs of people with multiple chronic conditions or frail elderly - and mapping workflow of 3 care managers with similar patients  Patients with complex needs: multiple chronic conditions or frail elderly | Functional, emotional, medical, and personal needs | N/A |
| Bridges, 2015^62^  UK | Clinicians at breast and colorectal cancer services directly involved in face-to-face care  Complex health and social care needs: older people with complex needs | N/A | Multidisciplinary team meetings are focused on cancer pathology rather than patient; information about co-morbidity not always included; rare to see complex patient-centered information in meetings; importance of patient input; national cancer targets dictate time between referral, diagnosis, and treatment; large # of patient cases to review impacted team meetings and led to quick decisions; desire to involve patients in decision-making but not always enough time for it, especially with older persons due to busy clinic schedule; need to form trusting relationship; pertinent patient information not always available at treatment-decision time. |
| Das, 2019^63^  USA | 21 high-need high-cost patients and 3 primary caregivers representing an urban health care system in New York City and a second one in Gainesville, FL  High-need, high-cost: at least 1 chronic medical condition and either 3 or more ED visits or 2 or more inpatient admissions during the 6 months prior to initiation of the study | Care management; readily avilable at-home physical therapy & nursing services; home delivery of prescription medications & easier reiflls; telemedicine; more after-hour clinics | N/A |
| Goodridge, 2019^64^  Canada | 4 patients, 2 managers, 1 social work case manager, 5 nurse clinicians  Socially complex patients: patients with chronic obstructive pulmonary disease who were eligible, but declined enrollment in a traditional chronic disease management program | From patient perspective - Poverty (inadequate housing, missed appointments, medication), disability (physical health in combination with social and/or mental health problems, low health literacy), personal attitudes and beliefs (shame, lack of trust in others). From provider perspective - Availability (not meeting needs of users); accessibility (lack of transportation, poor communication), acceptability (trust) | N/A |
| Kuluski, 2017^65^  Canada | 24 care providers working in health and social care  Complex care needs: characterized by multimorbidity, mental health challenges and social deprivation | N/A | Relationships as foundation of care; desired processes and structures for care; barriers and workarounds for desired care |
| Larsson, 2017^66^  Sweden | 18 primary care managers in western Sweden  Multimorbidity: having 2 or more chronic medical conditions | Care coordination for regular contact with and treatment from various healthcare providers | Perception of care planning (approach, planning, practice, & roles); cumulative assessment of cooperation with others |
| Maneze, 2014^67^  Australia | 13 patients with type 2 diabetes (10 with concomitant chronic condition), mostly socioeconomically disadvantaged  Complex needs: patients with type 2 diabetes and concomitant chronic condition, mostly socioeconomically disadvantaged | Need for better coordinated care (e.g., inconvenience of multiple health professionals, increased medications, travel); higher perceived cost due to multiple referrals so fewer follow-up visits; difficulty in managing medical needs as well as socioeconomic and cultural concerns (should be viewed as one patient with multiple conditions versus having each co-morbid condition managed by a separate health professional); poor understanding of their diabetes and self-management; lack of communication among healt professionals; important resource is carer as coordinator | N/A |
| Mautner, 2013^68^  USA | 19 high-utilizer patients (Camden Coalition)  High utilizer: patients that use high volumes of hospital-based health services | Themes: patients experienced childhood instability; patients had difficult relationships with healthcare system which included negative interactions with providers; patients endorsed importance of positive relationships with providers and care management team | N/A |
| Osborn, 2015^69^  Multiple countries | 11,547 primary care physicians from 10 countries  Complex health needs: aging patients with complex care needs (chronic care management, community and social services, episodic acute care) | N/A | Self-management support; clinical information systems; delivery system redesign (such as multidisciplinary teams); decision support; healthcare organization drivers (such as incentives & leadership); community resources |
| Perkerson, 2001^70^  USA | 122 patients in the two highest risk groups (Duke Case Mix System Classes 3 & 4)  Potential high utilizers: Duke Case Mix System Class 3 or 4 (highest predicted charges) patients | Main health problems being experienced; sources of the most help; main difficulties in getting help; what more the DFMC could do to help; type of possible help programs;mose useful things to discuss in help sessions; whether they might participate in help programs | N/A |
| Poitras, 2019^71^  Canada | 16 patients, 38 clinicians, 6 case managers, 14 decision-makers  Complex care needs: diagnosed with at least 1 chronic condition and identified as a frequent user of health care services by a program or health care providers | Service utilization and provider choice; global management of the physical & social environment; level of care & end of life; management of the health condition; acceptance of the health condition | Need more time with patients; would like to be better equipped for presenting options and integrating interprofessional perspective into shared decision making; need more training; need to provide the right tools to the professionals; administrative burden |
| Pratt, 2006^72^  UK | 6 district nurses  Highly complex patients: patients with long-term condition | N/A | Role definition; recognizing and developing skills; education for biomedical approach (work-based learning) |
| Ryan, 2016^73^  USA | 3,009 U.S. adults (1,805 high-need adults & 1,204 other adults without high needs)  High-need: adults with 2 or more major chronic conditions; may or may not have functional limitations in ability to perform daily tasks; some under age 65 with disability; elderly with multiple functional limitations | Health care utilization; access barriers; promising interventions (e.g., regular doctor or place of care, after-hours care); person-centered communication | N/A |
| Salzberg, 2016^74^  USA | Noninstitutionalized civilian US adults 18 years and older; about 12 million  High-need patient: 3 or more chronic conditions and a functional limitation in their ability to care for themselves or perform routine daily tasks | 1 in 5 high-need adults reported having an unmet medical need (forgoing or delaying needed medical care or prescription medication in the past year) | N/A |
| Schoen, 2011^75^  Schoen, 2008^76^  Multiple countries | 18,667 patients with serious illnesses, serioius injuries, or chronic diseases in 11 countries  Sicker adults: complex chronic conditions and recent extensive use of the health system | Findings highlight the need for system innovations to improve outcomes for patients with complex chronic conditions | N/A |
| Soto Mas, 2019^77^  USA | 48 participants with complex conditions  Complex conditions: patients in the top 1% of costliest and complex users based on claims and diagnosis/prognosis | Lack of economic resources to pay for healthcare and housing, lack of transportation, insurance, health literacy, access to care, access to food and other socioeconomic needs | N/A |
| Steele Gray, 2014^78^  Canada | Community dwelling individuals (10 patients, 2 caregivers, 2 who were both patient and caregiver)  Complex chronic disease and disability: 2 or more chronic illness and symptoms that have an impact on their daily living (resulting in using more care, experiencing por care coordination, & having a higher risk of poor health outcomes vs single illnesses | Patient-provider interactions (open, ongoing, 2-way communication; timely feedback; high quality); coordination of provider to provider interactions; access to needed health care services, specialists, and treatments (medications); patient-centered approach to care | N/A |
| Whitebird, 2017^79^  USA | 1554 primary care clinicians  Complex patients: patients with depression and diabetes and/or cardiovascular disease | N/A | Access to speciality care, patient attitudes, limited clinic resources, co-management of patients |
| Zulman, 2014^80^  Hsu, 2019^81^  USA | High-needs patients enrolled in VA intensive outpatient care programs  High-need, high-cost: 5% most costly patients in fiscal year | Medical neighborhood, medical status, social support, self-management/mental health (specifically direct/extended hour access, care coordination, intensive management, social work services, recreational services) | N/A |

## **Appendix Table 6: Literature Review Evidence Table Intervention Taxonomy**

| **Author, year** | **Country**  **Design** | **Definition** | **Taxonomy domains** |
| --- | --- | --- | --- |
| Anderson, 2015^82^  Thurber, 2019^83^ | USA  Literature review | People with high needs and high costs | Ability to target high-cost, high-need patients  Environment for successful leadership at all levels  Program size facilitates communication  Reflection of local and changing circumstances  Feedback to clinicians and care coordinators  Effective interactions with patients  Focus on care transitions  Programs to reduce workload of physicians |
| Bleich, 2015^2^ | USA  Systematic review | At least 2 chronic conditions | Care or case management  Chronic disease self-management  Disease management  Nursing home  Transitional care (e.g., hospital to home) |
| Boult, 2009^4^ | USA  Literature review | Chronically ill older persons | Interdisciplinary primary care  Care and case management  Disease management  Preventive home visits  Comprehensive geriatric assessment, geriatric evaluation and management  Pharmaceutical care  Chronic disease self-management  Proactive rehabilitation  Caregiver education and support  Transitional care  Substitute hospital at home  Early discharge hospital at home  Care in nursing homes  Prevention and management of delirium  Comprehensive inpatient care |
| Coller, 2014^7^ | USA  Systematic review | Children with chronic or complex illness | Patient/family behavior focus  Patient/family characteristics focus  Health care professional focus  Health system focus |
| Edwards, 2017^11^ | USA  Systematic review | Patients identified as high risk for hospital admission or death | Primary care replacement home-based care  Primary care replacement clinic-based case  Primary care augmentation (e.g., adding an interdisciplinary team) |
| Hong, 2014^84^ | USA  Literature review | Multiple or complex conditions, often combined with behavioral health problems or socioeconomic challenges | Customize approach to local contexts and caseloads  Use a combination of qualitative and quantitative methods to identify patients  Consider care coordination one of a key roles (ensure professionals share information, secure smooth referrals, help patients find resources in health systems and communities)  Focus on building trusting relationships with patients as well as their primary care professionals  Match team composition and interventions to patient needs  Offer specialized training for team members  Use health information technology to bolster efforts |
| Hudon, 2019^17^ | Multiple countries  Systematic review | Frequent healthcare use (using a threshold of number of health care visits) | High intensity case management  Multidisciplinary / interorganizational care plan |
| Kastner, 2018^20^ | Multiple countries  Systematic review | Older adults with multiple chronic diseases | Coordination of care  Cognitive-behavioral  Information and communication technology-based  Self-management |
| Long, 2017^85^  Figueroa, 2018^86^ | USA  Literature review | Clinical and functional groups (children with complex needs; non-elderly, disabled; multiple chronic; major complex chronic; frail elderly; advancing illness)  Behavioral and social assessment (behavioral health factors; social risk factors): substance abu | Care and condition attributes: assessment, targeting, planning, alignment, training, communication, monitoring, continuity  Delivery features: teamwork, coordination, responsiveness, feedback, medication management, outreach, integration, follow up |
| McCarthy, 2015^87^ | USA  Review of reviews | Multiple chronic health conditions and/or functional limitations; possible unmet social needs; poorly service by current health care delivery and financing arrangments | Content/features: Targeting individuals most likely to benefit, comprehensive assessment, care planning and patient monitoring, engagement in patient self-care, coordination, facilitation of transition and community resources, appropriate care  Execution/methods: Interdisciplinary teamwork, care manager rapport building, coaching and behavior-change techniques, standardized processes, effective use of health IT, outcomes measurement to evaluate and improve |
| Poitras, 2018^30^ | Multiple countries  Scoping review | Multi-morbidity: presence of multiple chronic or long-term conditions that can include both physical and mental disease | Patient-oriented interventions: providing patient oriented approach, supporting self-management  Professional interventions: training healthcare professionals  Organizational interventions: enhancing interdisciplinary team approach, supporting decision process and evidence-based practice, providing case/care management, integrating information technology |
| Smith, 2012^36^  Smith, 2017^37^; Smith, 2016^38^ | USA  Systematic review | Multi-morbidity: 2+ chronic conditions in an individual | Patient-oriented interventions  Organizational interventions |
| Soril, 2015^40^ | Multiple countries  Systematic review | Frequent emergency department use | Case management  Individualized care plans  Information sharing |
| Zulman, 2014^80^  Hsu, 2019^81^ | USA  Cohort study | Outpatients with total healthcare costs in the top 5% for the facility or 1-year hospitalization risk in the top 5% | Medical patient goals  Behavioral patient goals  Social patient goals |

## **Panel Composition and Stakeholder Representation**

- Lieutenant Colonel Ben Dennis, U.S. Army Retired (Patient representative): Chairman for the Veterans Affairs Greater Los Angeles Healthcare System Veterans Patient Advocacy Council
- Varis Green (Patient representative): U.S. Army National Guard, Honorably Discharged
- Samuel Edwards, MD, MPH (Primary care physician representative): Veterans Affairs Portland Health Care System; Oregon Health & Science University
- Robert Carr, RN (Nursing representative): Greater Los Angeles Greater Health Care System Veterans Affairs
- Nadereh Pourat, PhD (Policy representative): University of California, Los Angeles (UCLA) Center for Health Policy Research; University of California, Los Angeles Fielding School of Public Health and University of California, Los Angeles School of Dentistry
- Robert Friedman, PhD, LCSW (Social work representative): Veterans Affairs Greater Los Angeles Healthcare System
- James Zenner, U.S. Army Veteran (Peer navigation (community) representative): Los Angeles County Department of Mental Health
- Donna Zulman, MD, MS (Improvement implementation representative): Stanford University and Veterans Affairs Palo Alto Healthcare System
- Bowen Chung, MD, MSHS (Patient population mental health representative): Department of Psychiatry and Biobehavioral Sciences at the David Geffen School of Medicine at UCLA; RAND Corporation; Los Angeles Biomedical Research Institute, and Healthy African American Families II
- Sonya Gabrielian, MD, MPH (Patient population homelessness representative): Veterans Affairs Greater Los Angeles and University of California Los Angeles David Geffen School of Medicine
- David Ganz, MD, PhD (Patient population older adults representative): VA Greater Los Angeles Healthcare System’s Geriatric Research, Education and Clinical Center (GRECC) and the Center for the Study of Healthcare Innovation, Implementation and Policy (CSHIIP); University of California, Los Angeles; RAND Corporation
- Laura-Mae Baldwin, MD, PhD (Patient population addiction representative): University of Washington's Department of Family Medicine

## **Appendix Table 7: Stakeholder Endorsement of Terminology**

| **High Need Description** | **Mean (SD)** | **Mode** | **Number of endorsements for category 5** |
| --- | --- | --- | --- |
| High utilizer (services) | 4.45 (0.66) | 5 | 55% |
| Complex | 4.09 (0.90) | 5 | 45% |
| Multi-morbid | 4.00 (0.85) | 5 | 36% |
| High risk | 4.18 (0.72) | 4 | 36% |
| Vulnerable | 3.73 (1.14) | 4 | 27% |
| Medically complex | 3.91 (0.79) | 3, 4 | 27% |
| High cost | 3.64 (1.07) | 3, 4 | 27% |
| High utilizers (cost) | 3.73 (0.86) | 4 | 18% |
| Super user | 3.09 (1.50) | 4 | 18% |

Notes: Scale from 1 (not relevant) to 5 (captures high need patients really well); the table is ordered by number of endorsements for category 5, the darkest shade indicates the item’s mean was consistent with the highest answer category

## **Appendix Table 8: Stakeholder Endorsement of Statements Uniqueness vs Communalities**

| **High Need Description** | **Mean (SD)** | **Mode** | **Number of endorsements for category 5** |
| --- | --- | --- | --- |
| Patients' needs are unique but there are some areas of commonality for high need patients | 4.45 (0.66) | 4 | 55% |
| When determining needs of patients, it is important to differentiate subgroups of high need patients | 4.27 (0.75) | 5 | 45% |
| There are recommendations for healthcare delivery organizations that can be made regarding the identification of high need patients | 4.09 (0.67) | 4 | 27% |
| There are recommendations for healthcare delivery organizations that can be made regarding interventions for high need patients | 4.09 (0.67) | 4 | 27% |
| There are several interventions and tools that would be helpful for high need patients regardless of the individual needs | 4.00 (0.74) | 4 | 27% |
| It is important for healthcare delivery organizations to differentiate subgroups of patients because the approaches will vary considerably depending on the patient subgroup | 3.91 (0.79) | 3, 4 | 27% |
| To support healthcare providers caring for complex/high need patients, it is important to differentiate subgroups of patients because the needs of healthcare providers may vary considerably depending on the subgroup | 3.73 (0.96) | 3 | 27% |
| There is sufficient overlap across subgroups and across individuals to make recommendations for healthcare delivery organizations | 3.70 (0.78) | 4 | 10% |
| There is sufficient overlap across patient subgroups and across individuals to make recommendations regarding the care of high need patients | 3.55 (0.89) | 4 | 9% |
| There is sufficient overlap across patient subgroups and across individuals to make recommendations regarding the care of high need patients | 3.55 (0.89) | 4 | 9% |
| There is sufficient overlap across high need patients to make recommendations for healthcare providers | 3.64 (0.88) | 4 | 9% |
| Patients' needs are unique, and it is unlikely that there are interventions or tools that are helpful for all high need patients | 2.82 (0.94) | 3 | 0% |

Notes: Scale from 1 (strongly disagree) to 5 (strongly agree); the table is ordered by the number of endorsements for category 5, the darkest shade indicates the item’s mean was consistent with the highest answer category

## **Appendix Figure 2: Need of High-Needs Patients, Their Care Professionals, and Care Organizations**


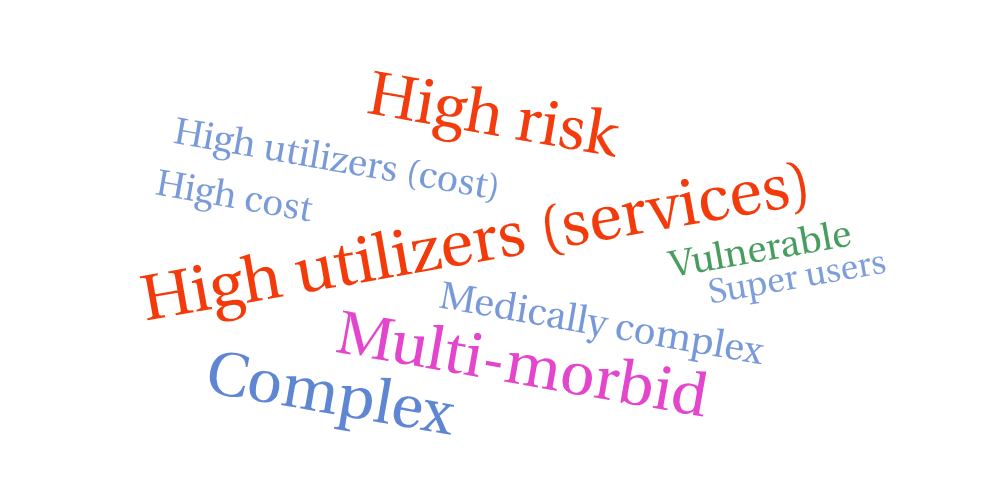


Note: Size of terms proportionate to the number of times the term was selected as an essential description of high need patients

## **Appendix Table 9: Stakeholder Endorsement of Needs**

| **Domain** | **Mean (SD)** | **Mode** | **Endorsement as ‘essential’** |
| --- | --- | --- | --- |
| **Patient** |  |  |  |
| Social circumstances (e.g., homelessness) | 5.00 (0.00) | 5 | 100% |
| Medical complexity of the diagnosis | 4.73 (0.62) | 5 | 82% |
| Functional (e.g., missing appointments) | 4.73 (0.45) | 5 | 73% |
| Trust (in healthcare professionals and organization) | 4.67 (0.60) | 5 | 73% |
| Interpersonal (e.g., support from family) | 4.64 (0.64) | 5 | 73% |
| Care access | 4.60 (0.71) | 5 | 73% |
| Organizational navigation | 4.55 (0.78) | 5 | 73% |
| Behavioral (e.g., following recommendations) | 4.55 (0.50) | 5 | 55% |
| Cognitive (e.g., understanding care sequence steps) | 4.55 (0.75) | 5 | 55% |
| Psychiatric complexity | 4.33 (1.07) | 5 | 60% |
| Psychosocial (e.g., interaction with professionals and others) | 3.73 (1.29) | 5 | 40% |
| Frequent contact, frequent use | 3.33 (1.33) | 4 | 22% |
| Multi-morbidity | 3.40 (1.20) | 4 | 20% |
| Practical-care related (e.g., transportation) | 3.30 (1.19) | 2 | 20% |
| Care arrangement (intensity, help from case manager) | 3.20 (1.33) | 4 | 20% |
| Intense appropriate service use | 3.00 (1.34) | 2 | 20% |
| Patient cost | 2.90 (1.14) | 2 | 20% |
| Inappropriate service use (e.g., primary care in the ER) | 3.10 (1.22) | 4 | 10% |
| Organizational cost (incurring through the patient) | 2.60 (0.80) | 2 | 0% |
| Knowledge and health literacy | 3.00 (1.00) | 4 | 0% |
| Chronicity (of conditions) | 3.10 (1.04) | 4 | 0% |
| **Professionals** |  |  |  |
| Communication (e.g., between healthcare professionals) | 4.90 (0.26) | 5 | 93% |
| Interdisciplinary care team | 4.80 (0.31) | 5 | 80% |
| Time (e.g., needed for additional care arrangements) | 4.67 (0.62) | 5 | 73% |
| Provider skills | 4.60 (0.63) | 5 | 67% |
| Provider knowledge about care and system | 3.60 (1.28) | 5 | 40% |
| Care arrangement (e.g., case management) | 3.30 (1.42) | 2 | 40% |
| Provider system needs (panel size adjustment) | 3.40 (1.20) | 2, 3, 5 | 30% |
| Team characteristics (e.g., well-coordinated team) | 3.40 (1.28) | 2 | 30% |
| Provider health and wellbeing | 3.10 (0.94) | 3 | 10% |
| Information need about the patient | 2.90 (0.94) | 2, 3 | 10% |
| **Organization** |  |  |  |
| Staffing arrangements (e.g., multi-disciplinary teams) | 4.30 (0.90) | 5 | 50% |
| Collaboration within and outside the healthcare system | 4.30 (0.90) | 5 | 50% |
| Care approach and culture | 4.20 (0.98) | 5 | 50% |
| Technology (e.g., IT support for information exchange) | 3.90 (1.04) | 4 | 40% |
| Financial (e.g., resources available to care for complex patients) | 3.70 (1.00) | 3 | 30% |
| Investment in workforce (e.g., training) | 3.70 (1.00) | 3 | 30% |
| Expertise (e.g., social worker is part of the care team) | 3.60 (1.11) | 3, 5 | 30% |
| Measurement (e.g., to identify high-need patients) | 3.50 (0.81) | 3 | 10% |

Notes: Scale from 1 (not relevant) to 5 (essential); ER emergency room, IT information technology; the table is ordered by the number of endorsements for category 5, the darkest shade indicates the item’s mean was consistent with a rating of essential, a lighter shade indicates the mean was consistent with a rating of important

## **Appendix Table 10: Stakeholder Endorsement of Intervention Categories**

| **Intervention Domain** | **Directed at** | **Mean (SD)** | **Mode** | **Number of endorsements as ‘essential’** |
| --- | --- | --- | --- | --- |
| Organizational Interventions to address care coordination  (e.g., establishing a care manager) | Organization | 4.60 (0.66) | 5 | 70% |
| Organizational interventions to identify high need patients  (e.g., tracking visits) | Organization | 4.70 (0.46) | 5 | 70% |
| Structural interventions  (e.g., establish regular telephone contact schedules) | Organization | 4.50 (0.67) | 5 | 60% |
| Interventions to address social support of patients | Patient | 4.50 (0.67) | 5 | 60% |
| Practical support interventions for receiving care  (e.g., transportation voucher to enable practice visit) | Patient | 4.60 (0.49) | 5 | 60% |
| Practical support interventions for potentially reducing the need for care (e.g., housing, food bank) | Patient | 4.60 (0.49) | 5 | 60% |
| Patient interventions to address care navigation (e.g., health literacy) | Patient | 4.50 (0.67) | 5 | 60% |
| Organizational interventions  (changing procedures) | Organization | 4.50 (0.50) | 4, 5 | 50% |
| Interventions to address patient characteristics  (e.g., interventions primarily addressing coping, not treatment) | Patient | 4.44 (0.68) | 5 | 56% |
| Organizational interventions to address medical complexities  (e.g., interdisciplinary care) | Organization | 4.20 (0.98) | 4 | 50% |
| Interventions to address medical complexities of the diagnosis | Patient | 4.20 (0.98) | 5 | 50% |
| Patient-oriented interventions  (e.g., changing patient behavior) | Patient | 4.30 (0.64) | 4 | 40% |
| Educational Interventions for patients  (self-management support, patient education) | Patient | 4.20 (0.75) | 4, 5 | 40% |
| Professional interventions focused on medical complexity of multi-morbidity (e.g., reviewing drug interactions) | Professional | 4.00 (0.89) | 4 | 30% |
| Professional-oriented interventions  (e.g., changing provider behavior) | Professional | 3.90 (0.94) | 4 | 30% |

Notes: Scale from 1 (not relevant / not important) to 5 (essential); the table is ordered by the number of endorsements for category 5, the darkest shade indicates the item’s mean was consistent with a rating of essential, a lighter shade indicates the mean was consistent with a rating of important

## **References**

1. Baker, JM, Grant, RW, and Gopalan, A.“A systematic review of care management interventions targeting multimorbidity and high care utilization.” *BMC Health Serv Res*. 2018;18(1):65.

2. Bleich, SN, Sherrod, C, Chiang, A, Boyd, C, Wolff, J, DuGoff, E, Salzberg, C, Anderson, K, Leff, B, and Anderson, G.“Systematic Review of Programs Treating High-Need and High-Cost People With Multiple Chronic Diseases or Disabilities in the United States, 2008-2014.” *Prev Chronic Dis*. 2015;12:E197.

3. Boehmer, KR, Abu Dabrh, AM, Gionfriddo, MR, Erwin, P, and Montori, VM.“Does the chronic care model meet the emerging needs of people living with multimorbidity? A systematic review and thematic synthesis.” *PLoS One*. 2018;13(2):e0190852.

4. Boult, C, Green, AF, Boult, LB, Pacala, JT, Snyder, C, and Leff, B.“Successful models of comprehensive care for older adults with chronic conditions: evidence for the Institute of Medicine's "retooling for an aging America" report.” *J Am Geriatr Soc*. 2009;57(12):2328-37.

5. Bunn, F, Goodman, C, Russell, B, Wilson, P, Manthorpe, J, Rait, G, Hodkinson, I, and Durand, MA.“Supporting shared decision making for older people with multiple health and social care needs: a realist synthesis.” *BMC Geriatr*. 2018;18(1):165.

6. Butterworth, JE, Hays, R, McDonagh, ST, Richards, SH, Bower, P, and Campbell, J.“Interventions for involving older patients with multi-morbidity in decision-making during primary care consultations.” *Cochrane Database Syst Rev*. 2019;2019(10).

7. Coller, RJ, Nelson, BB, Sklansky, DJ, Saenz, AA, Klitzner, TS, Lerner, CF, and Chung, PJ.“Preventing hospitalizations in children with medical complexity: a systematic review.” *Pediatrics*. 2014;134(6):e1628-47.

8. Coventry, PA, Small, N, Panagioti, M, Adeyemi, I, and Bee, P.“Living with complexity; marshalling resources: a systematic review and qualitative meta-synthesis of lived experience of mental and physical multimorbidity.” *BMC Fam Pract*. 2015;16:171.

9. De Groot , V, Beckerman, H, Lankhorst, GJ, and Bouter, LM.“How to measure comorbidity: a critical review of available methods.” *J Clin Epidemiol* 2003;56:221–9.

10. Diederichs, C, Berger, K, and Bartels, DB.“The Measurement of Multiple Chronic Diseases-A Systematic Review on Existing Multimorbidity Indices.” *Journals of Gerontology Series a-Biological Sciences and Medical Sciences*. 2011;66(3):301-11.

11. Edwards, ST, Peterson, K, Chan, B, Anderson, J, and Helfand, M.“Effectiveness of Intensive Primary Care Interventions: A Systematic Review.” *J Gen Intern Med*. 2017;32(12):1377-86.

12. Fraccaro, P, Arguello Casteleiro, M, Ainsworth, J, and Buchan, I.“Adoption of clinical decision support in multimorbidity: a systematic review.” *JMIR Med Inform*. 2015;3(1):e4.

13. Gobeil-Lavoie, AP, Chouinard, MC, Danish, A, and Hudon, C.“Characteristics of self-management among patients with complex health needs: a thematic analysis review.” *BMJ Open*. 2019;9(5):e028344.

14. Haroun, D, Smits, F, van Etten-Jamaludin, F, Schene, A, van Weert, H, and Ter Riet, G.“The effects of interventions on quality of life, morbidity and consultation frequency in frequent attenders in primary care: A systematic review.” *Eur J Gen Pract*. 2016;22(2):71-82.

15. Hohmann, NS, McDaniel, CC, Mason, SW, Cheung, WY, Williams, MS, Salvador, C, Graves, EK, Camp, CN, and Chou, C.“Patient perspectives on primary care and oncology care coordination in the context of multiple chronic conditions: A systematic review.” *Res Social Adm Pharm*. 2019.

16. Hohmann, NS, McDaniel, CC, Mason, SW, Cheung, WY, Williams, MS, Salvador, C, Graves, EK, Camp, CN, and Chou, CH.“Healthcare providers' perspectives on care coordination for adults with cancer and multiple chronic conditions: a systematic review.” *Journal of Pharmaceutical Health Services Research*.

17. Hudon, C, Chouinard, MC, Pluye, P, El Sherif, R, Bush, PL, Rihoux, B, Poitras, ME, Lambert, M, Zomahoun, HTV, and Legare, F.“Characteristics of Case Management in Primary Care Associated With Positive Outcomes for Frequent Users of Health Care: A Systematic Review.” *Ann Fam Med*. 2019;17(5):448-58.

18. Huntley, AL, Johnson, R, Purdy, S, Valderas, JM, and Salisbury, C.“Measures of multimorbidity and morbidity burden for use in primary care and community settings: a systematic review and guide.” *Ann Fam Med*. 2012;10(2):134-41.

19. Johnston, MC, Crilly, M, Black, C, Prescott, GJ, and Mercer, SW.“Defining and measuring multimorbidity: a systematic review of systematic reviews.” *Eur J Public Health*. 2019;29(1):182-89.

20. Kastner, M, Cardoso, R, Lai, Y, Treister, V, Hamid, JS, Hayden, L, Wong, G, Ivers, NM, Liu, B, Marr, S, Holroyd-Leduc, J, and Straus, SE.“Effectiveness of interventions for managing multiple high-burden chronic diseases in older adults: a systematic review and meta-analysis.” *Cmaj*. 2018;190(34):E1004-e12.

21. Latour, CH, van der Windt, DA, de Jonge, P, Riphagen, II, de Vos, R, Huyse, FJ, and Stalman, WA.“Nurse-led case management for ambulatory complex patients in general health care: a systematic review.” *J Psychosom Res*. 2007;62(3):385-95.

22. Le Reste, JY, Nabbe, P, Manceau, B, Lygidakis, C, Doerr, C, Lingner, H, Czachowski, S, Munoz, M, Argyriadou, S, Claveria, A, Le Floch, B, Barais, M, Bower, P, Van Marwijk, H, Van Royen, P, and Lietard, C.“The European General Practice Research Network presents a comprehensive definition of multimorbidity in family medicine and long term care, following a systematic review of relevant literature.” *J Am Med Dir Assoc*. 2013;14(5):319-25.

23. Liddy, C, Blazkho, V, and Mill, K.“Challenges of self-management when living with multiple chronic conditions Systematic review of the qualitative literature.” *Canadian Family Physician*. 2014;60(12):1123-33.

24. Mangin, D, Stephen, G, Bismah, V, and Risdon, C.“Making patient values visible in healthcare: a systematic review of tools to assess patient treatment priorities and preferences in the context of multimorbidity.” *BMJ Open*. 2016;6(6):e010903.

25. Marcoux, V, Chouinard, MC, Diadiou, F, Dufour, I, and Hudon, C.“Screening tools to identify patients with complex health needs at risk of high use of health care services: A scoping review.” *PLoS One*. 2017;12(11):e0188663.

26. Marengoni, A, Angleman, S, Melis, R, Mangialasche, F, Karp, A, Garmen, A, Meinow, B, and Fratiglioni, L.“Aging with multimorbidity: A systematic review of the literature.” *Ageing Research Reviews*. 2011;10(4):430-39.

27. Moe, J, Kirkland, SW, Rawe, E, Ospina, MB, Vandermeer, B, Campbell, S, and Rowe, BH.“Effectiveness of Interventions to Decrease Emergency Department Visits by Adult Frequent Users: A Systematic Review.” *Acad Emerg Med*. 2017;24(1):40-52.

28. Ng, SK, Tawiah, R, Sawyer, M, and Scuffham, P.“Patterns of multimorbid health conditions: a systematic review of analytical methods and comparison analysis.” *International Journal of Epidemiology*. 2018;47(5):1687-704.

29. Pillay, M, Dennis, S, and Harris, MF.“Quality of care measures in multimorbidity.” *Aust Fam Physician*. 2014;43(3):132-6.

30. Poitras, ME, Maltais, ME, Bestard-Denomme, L, Stewart, M, and Fortin, M.“What are the effective elements in patient-centered and multimorbidity care? A scoping review.” *BMC Health Serv Res*. 2018;18(1):446.

31. Rosbach, M and Andersen, JS.“Patient-experienced burden of treatment in patients with multimorbidity - A systematic review of qualitative data.” *PLoS One*. 2017;12(6).

32. Sasseville, M, Chouinard, M-C, and Fortin, M.“Patient-reported outcomes in multimorbidity intervention research: A scoping review.” *International Journal of Nursing Studies*. 2018;77:145-53.

33. Singh, K, Drouin, K, Newmark, LP, Filkins, M, Silvers, E, Bain, PA, Zulman, DM, Lee, JH, Rozenblum, R, Pabo, E, Landman, A, Klinger, EV, and Bates, DW.“Patient-Facing Mobile Apps to Treat High-Need, High-Cost Populations: A Scoping Review.” *JMIR Mhealth Uhealth*. 2016;4(4):e136.

34. Sinnott, C, Mercer, SW, Payne, RA, Duerden, M, Bradley, CP, and Byrne, M.“Improving medication management in multimorbidity: development of the MultimorbiditY COllaborative Medication Review And DEcision Making (MY COMRADE) intervention using the Behaviour Change Wheel.” *Implement Sci*. 2015;10:132.

35. Sinnott, C, Mc Hugh, S, Browne, J, and Bradley, C.“GPs' perspectives on the management of patients with multimorbidity: systematic review and synthesis of qualitative research.” *Bmj Open*. 2013;3(9).

36. Smith, SM, Soubhi, H, Fortin, M, Hudon, C, and O'Dowd, T.“Managing patients with multimorbidity: systematic review of interventions in primary care and community settings.” *BMJ*. 2012;345:e5205.

37. Smith, SM, Soubhi, H, Fortin, M, Hudon, C, and O'Dowd, T.“Interventions for improving outcomes in patients with multimorbidity in primary care and community settings.” *Cochrane Database Syst Rev*. 2012;(4):Cd006560.

38. Smith, SM, Wallace, E, O'Dowd, T, and Fortin, M.“Interventions for improving outcomes in patients with multimorbidity in primary care and community settings.” *Cochrane Database Syst Rev*. 2016;3:Cd006560.

39. Smits, FT, Wittkampf, KA, Schene, AH, Bindels, PJ, and Van Weert, HC.“Interventions on frequent attenders in primary care. A systematic literature review.” *Scand J Prim Health Care*. 2008;26(2):111-6.

40. Soril, LJ, Leggett, LE, Lorenzetti, DL, Noseworthy, TW, and Clement, FM.“Reducing frequent visits to the emergency department: a systematic review of interventions.” *PLoS One*. 2015;10(4):e0123660.

41. Soril, LJ, Leggett, LE, Lorenzetti, DL, Noseworthy, TW, and Clement, FM.“Characteristics of frequent users of the emergency department in the general adult population: A systematic review of international healthcare systems.” *Health Policy*. 2016;120(5):452-61.

42. Stokes, J, Panagioti, M, Alam, R, Checkland, K, Cheraghi-Sohi, S, and Bower, P.“Effectiveness of Case Management for 'At Risk' Patients in Primary Care: A Systematic Review and Meta-Analysis.” *PLoS One*. 2015;10(7).

43. Vermunt, N, Harmsen, M, Westert, GP, Olde Rikkert, MGM, and Faber, MJ.“Collaborative goal setting with elderly patients with chronic disease or multimorbidity: a systematic review.” *BMC Geriatr*. 2017;17(1):167.

44. Violan, C, Foguet-Boreu, Q, Flores-Mateo, G, Salisbury, C, Blom, J, Freitag, M, Glynn, L, Muth, C, and Valderas, JM.“Prevalence, determinants and patterns of multimorbidity in primary care: a systematic review of observational studies.” *PLoS One*. 2014;9(7):e102149.

45. Welzel, FD, Stein, J, Hajek, A, Konig, HH, and Riedel-Heller, SG.“Frequent attenders in late life in primary care: a systematic review of European studies.” *BMC Fam Pract*. 2017;18(1):104.

46. Willadsen, TG, Bebe, A, Koster-Rasmussen, R, Jarbol, DE, Guassora, AD, Waldorff, FB, Reventlow, S, and Olivarius Nde, F.“The role of diseases, risk factors and symptoms in the definition of multimorbidity - a systematic review.” *Scand J Prim Health Care*. 2016;34(2):112-21.

47. Yardley, S, Cottrell, E, Rees, E, and Protheroe, J.“Modelling successful primary care for multimorbidity: a realist synthesis of successes and failures in concurrent learning and healthcare delivery.” *BMC Fam Pract*. 2015;16:23.

48. Yurkovich, M, Avina-Zubieta, JA, Thomas, J, Gorenchtein, M, and Lacaille, D.“A systematic review identifies valid comorbidity indices derived from administrative health data.” *J Clin Epidemiol*. 2015;68(1):3-14.

49. Zullig, LL, Whitson, HE, Hastings, SN, Beadles, C, Kravchenko, J, Akushevich, I, and Maciejewski, ML.“A Systematic Review of Conceptual Frameworks of Medical Complexity and New Model Development.” *J Gen Intern Med*. 2016;31(3):329-37.

50. Belanger, E, Silver, B, Meyers, DJ, Rahman, M, Kumar, A, Kosar, C, and Mor, V.“A Retrospective Study of Administrative Data to Identify High-Need Medicare Beneficiaries at Risk of Dying and Being Hospitalized.” *J Gen Intern Med*. 2019;34(3):405-11.

51. Buja, A, Claus, M, Perin, L, Rivera, M, Corti, MC, Avossa, F, Schievano, E, Rigon, S, Toffanin, R, Baldo, V, and Boccuzzo, G.“Multimorbidity patterns in high-need, high-cost elderly patients.” *PLoS One*. 2018;13(12):e0208875.

52. Hayes, SL, Salzberg, CA, McCarthy, D, Radley, DC, Abrams, MK, Shah, T, and Anderson, GF.“High-Need, High-Cost Patients: Who Are They and How Do They Use Health Care? A Population-Based Comparison of Demographics, Health Care Use, and Expenditures.” *Issue Brief (Commonw Fund)*. 2016;26:1-14.

53. Price-Haywood, EG, Petersen, H, Burton, J, Harden-Barrios, J, Adubato, M, Roberts, M, and Markward, N.“Outpatient Complex Case Management: Health System-Tailored Risk Stratification Taxonomy to Identify High-Cost, High-Need Patients.” *J Gen Intern Med*. 2018;33(11):1921-27.

54. Matzer, F, Wisiak, UV, Graninger, M, Sollner, W, Stilling, HP, Glawischnig-Goschnik, M, Lueger, A, and Fazekas, C.“Biopsychosocial health care needs at the emergency room: challenge of complexity.” *PLoS One*. 2012;7(8):e41775.

55. Nuti, SV, Doupe, P, Villanueva, B, Scarpa, J, Bruzelius, E, and Baum, A.“Characterizing Subgroups of High-Need, High-Cost Patients Based on Their Clinical Conditions: a Machine Learning-Based Analysis of Medicaid Claims Data.” *J Gen Intern Med*. 2019;34(8):1406-08.

56. Sarnak, DO and Ryan, J.“How High-Need Patients Experiences the Health Care System in Nine Countries.” *Issue Brief (Commonw Fund)*. 2016;1:1-14.

57. Osborn, R, Moulds, D, Squires, D, Doty, MM, and Anderson, C.“International survey of older adults finds shortcomings in access, coordination, and patient-centered care.” *Health Aff (Millwood)*. 2014;33(12):2247-55.

58. Schickedanz, A, Sharp, A, Hu, YR, Shah, NR, Adams, JL, Francis, D, and Rogers, A.“Impact of Social Needs Navigation on Utilization Among High Utilizers in a Large Integrated Health System: a Quasi-experimental Study.” *J Gen Intern Med*. 2019;34(11):2382-89.

59. Rogers, AJ, Hamity, C, Sharp, AL, Jackson, AH, and Schickedanz, AB.“Patients' Attitudes and Perceptions Regarding Social Needs Screening and Navigation: Multi-site Survey in a Large Integrated Health System.” *J Gen Intern Med*. 2020;35(5):1389-95.

60. Smeets, RGM, Elissen, AMJ, Kroese, M, Hameleers, N, and Ruwaard, D.“Identifying subgroups of high-need, high-cost, chronically ill patients in primary care: A latent class analysis.” *PLoS One*. 2020;15(1):e0228103.

61. Bhattacharyya, O, Mossman, K, Gustafsson, L, and Schneider, EC.“Using Human-Centered Design to Build a Digital Health Advisor for Patients With Complex Needs: Persona and Prototype Development.” *J Med Internet Res*. 2019;21(5):e10318.

62. Bridges, J, Hughes, J, Farrington, N, and Richardson, A.“Cancer treatment decision-making processes for older patients with complex needs: a qualitative study.” *BMJ Open*. 2015;5(12):e009674.

63. Das, LT, Abramson, EL, and Kaushal, R.“High-Need, High-Cost Patients Offer Solutions for Improving Their Care and Reducing Costs.” *NEJM Catal*. 2019;2019.

64. Goodridge, D, Bandara, T, Marciniuk, D, Hutchinson, S, Crossman, L, Kachur, B, Higgins, D, and Bennett, A.“Promoting chronic disease management in persons with complex social needs: A qualitative descriptive study.” *Chron Respir Dis*. 2019;16:1479973119832025.

65. Kuluski, K, Ho, JW, Hans, PK, and Nelson, M.“Community Care for People with Complex Care Needs: Bridging the Gap between Health and Social Care.” *Int J Integr Care*. 2017;17(4):2.

66. Larsson, LG, Back-Pettersson, S, Kylen, S, Marklund, B, and Carlstrom, E.“Primary care managers' perceptions of their capability in providing care planning to patients with complex needs.” *Health Policy*. 2017;121(1):58-65.

67. Maneze, D, Dennis, S, Chen, HY, Taggart, J, Vagholkar, S, Bunker, J, and Liaw, ST.“Multidisciplinary care: experience of patients with complex needs.” *Aust J Prim Health*. 2014;20(1):20-6.

68. Mautner, DB, Pang, H, Brenner, JC, Shea, JA, Gross, KS, Frasso, R, and Cannuscio, CC.“Generating hypotheses about care needs of high utilizers: lessons from patient interviews.” *Popul Health Manag*. 2013;16 Suppl 1:S26-33.

69. Osborn, R, Moulds, D, Schneider, EC, Doty, MM, Squires, D, and Sarnak, DO.“Primary Care Physicians In Ten Countries Report Challenges Caring For Patients With Complex Health Needs.” *Health Aff (Millwood)*. 2015;34(12):2104-12.

70. Parkerson, GR, Jr., Bell, HS, Albright, JB, and Krause, K.“A telephone needs assessment for potential high utilizers.” *Fam Med*. 2001;33(6):466-72.

71. Poitras, ME, Hudon, C, Godbout, I, Bujold, M, Pluye, P, Vaillancourt, VT, Debarges, B, Poirier, A, Prevost, K, Spence, C, and Legare, F.“Decisional needs assessment of patients with complex care needs in primary care.” *J Eval Clin Pract*. 2019.

72. Pratt, LR.“Long-term conditions 5: meeting the needs of highly complex patients.” *Br J Community Nurs*. 2006;11(6):234-5, 38-40.

73. Ryan, J, Abrams, MK, Doty, MM, Shah, T, and Schneider, EC.“How High-Need Patients Experience Health Care in the United States. Findings from the 2016 Commonwealth Fund Survey of High-Need Patients.” *Issue Brief (Commonw Fund)*. 2016;43:1-20.

74. Salzberg, CA, Hayes, SL, McCarthy, D, Radley, DC, Abrams, MK, Shah, T, and Anderson, GF.“Health System Performance for the High-Need Patient: A Look at Access to Care and Patient Care Experiences.” *Issue Brief (Commonw Fund)*. 2016;27:1-12.

75. Schoen, C, Osborn, R, Squires, D, Doty, M, Pierson, R, and Applebaum, S.“New 2011 survey of patients with complex care needs in eleven countries finds that care is often poorly coordinated.” *Health Aff (Millwood)*. 2011;30(12):2437-48.

76. Schoen, C, Osborn, R, How, SK, Doty, MM, and Peugh, J.“In chronic condition: experiences of patients with complex health care needs, in eight countries, 2008.” *Health Aff (Millwood)*. 2009;28(1):w1-16.

77. Soto Mas, F, Iriart, C, Pedroncelli, R, Binder, DS, Qualls, CR, and Price, B.“Impact of Health Care and Socioeconomic Needs on Health Care Utilization and Disease Management: The University of New Mexico Hospital Care One Program.” *Popul Health Manag*. 2019;22(2):113-19.

78. Steele Gray, C, Miller, D, Kuluski, K, and Cott, C.“Tying eHealth Tools to Patient Needs: Exploring the Use of eHealth for Community-Dwelling Patients With Complex Chronic Disease and Disability.” *JMIR Res Protoc*. 2014;3(4):e67.

79. Whitebird, RR, Solberg, LI, Crain, AL, Rossom, RC, Beck, A, Neely, C, Dreskin, M, and Coleman, KJ.“Clinician burnout and satisfaction with resources in caring for complex patients.” *General Hospital Psychiatry*. 2017;44:91-95.

80. Zulman, DM, Ezeji-Okoye, SC, Shaw, JG, Hummel, DL, Holloway, KS, Smither, SF, Breland, JY, Chardos, JF, Kirsh, S, Kahn, JS, and Asch, SM.“Partnered research in healthcare delivery redesign for high-need, high-cost patients: development and feasibility of an Intensive Management Patient-Aligned Care Team (ImPACT).” *J Gen Intern Med*. 2014;29 Suppl 4:861-9.

81. Hsu, KY, Slightam, C, Shaw, JG, Tierney, A, Hummel, DL, Goldstein, MK, Chang, ET, Boothroyd, D, and Zulman, DM.“High-Need Patients' Goals and Goal Progress in a Veterans Affairs Intensive Outpatient Care Program.” *J Gen Intern Med*. 2019;34(8):1564-70.

82. Anderson, GF, Ballreich, J, Bleich, S, Boyd, C, DuGoff, E, Leff, B, Salzburg, C, and Wolff, J.“Attributes common to programs that successfully treat high-need, high-cost individuals.” *Am J Manag Care*. 2015;21(11):e597-600.

83. Thurber, EG, Boyd, C, Leff, B, Wolff, J, and Anderson, G.“The Common Attributes of Successful Care Manager Programs for High-Need, High-Cost Persons: A Cross-Case Analysis.” *J Ambul Care Manage*. 2019;42(4):230-41.

84. Hong, CS, Siegel, AL, and Ferris, TG.“Caring for high-need, high-cost patients: what makes for a successful care management program?” *Issue Brief (Commonw Fund)*. 2014;19:1-19.

85. Long, P, Abrams, MK, Milstein, A, and et al. 2017. *Effective Care for High-Need Patients: Opportunities for Improving Outcomes, Value, and Health*. Washington, DC: National Academy of Medicine. 2017.

86. Figueroa, JF and Jha, AK.“Approach for Achieving Effective Care for High-Need Patients.” *JAMA Intern Med*. 2018;178(6):845-46.

87. McCarthy, D, Ryan, J, and Klein, S.“Models of Care for High-Need, High-Cost Patients: An Evidence Synthesis.” *Issue Brief (Commonw Fund)*. 2015;31:1-19.
